# Identification and Mechanism of Action of the Global Secondary Metabolism Regulator SaraC in *Stereum hirsutum*

**DOI:** 10.1128/spectrum.02624-22

**Published:** 2022-11-21

**Authors:** Qian-Yi Hu, Xue-Juan Pu, Guo-Hong Li, Chun-Qiang Li, Hong-Mei Lei, Ke-Qin Zhang, Pei-Ji Zhao

**Affiliations:** a State key Laboratory for Conservation and Utilization of Bio-Resources in Yunnan, School of Life Sciences, Yunnan University, Kunming, Yunnan, China; Westerdijk Fungal Biodiversity Institute

**Keywords:** DNA methylation, secondary metabolism, global regulator, methylome, transcriptome, metabolome

## Abstract

DNA methylation is an important factor in the regulation of gene expression. In analyzing genomic data of Stereum hirsutum FP-91666, we found a hypothetical bifunctional transcription regulator/O^6Me^guanine-DNA methyltransferase (named SaraC), which is widely present in both bacteria and fungi, and confirmed that its function in bacteria is mainly for DNA reparation. In this paper, we confirmed that SaraC has the function of DNA binding and demethylation through surface plasma resonance and reaction experiments *in vitro*. Then, we achieved the overexpression of *SaraC* (OES) in S. hirsutum, sequenced the methylation and transcription levels of the whole-genome, and further conducted untargeted metabolomics analyses of the OES transformants and the wild type (WT). The results confirmed that the overall-methylation levels of the transformants were significantly downregulated, and various genes related to secondary metabolism were upregulated. Through comparative untargeted metabolomic analyses, it showed that OES SA6 transformant produced a greater number of hybrid polyketides, and we identified 2 novel hybrid polyketides from the fermentation products of SA6. Our results show that overexpression *SaraC* can effectively stimulate the expression of secondary-metabolism-related genes, which could be a broad-spectrum tool for discovery of metabolites due to its cross-species conservation.

**IMPORTANCE** Fungi are one of the important sources of active compounds. However, in fungi, most of the secondary metabolic biosynthetic gene clusters are weakly expressed or silenced under conventional culture conditions. How to efficiently excavate potential new compounds contained in fungi is becoming a research hot spot in the world. In this study, we found a DNA demethylation protein (SaraC) and confirmed that it is a global secondary metabolism regulator in Stereum hirsutum FP-91666. In the past, SaraC-like proteins were mainly regarded as DNA repair proteins, but our findings proved that it will be a powerful tool for mining secondary metabolites for overexpression of SaraC, which can effectively stimulate the expression of genes related to secondary metabolism.

## INTRODUCTION

Fungi, as a secondary metabolite resource library, can produce many novel and active secondary metabolites (1, 2), which are a remarkably rich source of compounds with potential or actual medical value, such as antibacterial, anti-cancer, anti-malarial, and immune regulation, and have extremely high drug-development value (3, 4). With the rapid development of whole-genome sequencing technology and bioinformatics, genomic information of fungal genomes has revealed that the metabolic biosynthetic potential of most fungi has not yet been fully exploited. Many results show that the ability of fungi to encode secondary metabolic biosynthetic genes for metabolites is significantly greater than the sum of the metabolites found in these fungi (5, 6). The main reason for this may be due to silence or weak expression of metabolic biosynthetic genes under experimental conditions (7).

Given the importance of fungal secondary metabolites, there are numerous successful ways to mine potential products. How to activate silent gene clusters to obtain more active compounds with novel structures, and to tap and develop potential medicinal resources has become a trending research area. Specific transcription factors regulate one or several secondary metabolic pathways. Overexpression of certain pathway-specific regulatory factors is one of the effective methods to activate silent gene clusters. *AflR*, a Zn2Cys6-specific transcription factor located in the aflatoxin biosynthesis gene cluster of Aspergillus nidulans, has a significant effect on the synthesis of aflatoxin and sterigmatocystin after mutation or overexpression of *aflR* (8, 9). Subsequently, a silencing gene cluster, encoding hybrid PKS/NRPS, was successfully activated by inducing the expression of *apdR* and achieved aspyridones in A. nidulans (10). Many similar regulatory factors have been found in different filamentous fungi, such as MRTRI6 and Tri6, which regulate the biosynthesis of trichothecene in *Myrothecium roridum* and mycotoxin in Fusarium sporotrichioides, respectively (11, 12). Global regulatory factors could regulate various physiological functions and multiple secondary metabolic pathways of fungi (13). By far the most influential global regulator is *LaeA* and its transcription complex in fungi (14, 15). Knockout or overexpression of *LaeA* obviously affects the expression of secondary metabolic biosynthetic gene clusters (16, 17). LaeA forms a heterotrimeric complex with the fungus-conserved velvet subunits VeA and VelB; this heterotrimeric complex mediates the secondary metabolism and fungal development in A. nidulans (15).

In this study, when analyzing genomic data of Stereum hirsutum FP-91666 (wild type [WT]), we found a previously uncharacterized protein (GenBank: XP_007299133.1), which is a hypothetical, bifunctional transcription regulator/O6Meguanine-DNA methyltransferase, and it contains AdaA, Ada_Zn_binding and HTH_AraC domains. We have identified a globally positive transcription regulator in S. hirsutum FP-91666. Using a combination of heterologous expression, overexpression of *SaraC* in S. hirsutum, and methylome, transcriptome, metabolome, and metabolite isolation and identification, we have identified OES (overexpression of *SaraC*) transformants that activate expression of lots of secondary metabolic biosynthetic gene clusters. In addition, 2 novel hybrid polyketides were isolated and identified from the over-expressed transformants. SaraC-like proteins were mainly regarded as DNA repair proteins, but our findings shed light on that it will be a powerful tool for mining secondary metabolites for overexpression of *SaraC*, which can effectively stimulate the expression of genes related to secondary metabolism and it is widely distributed in fungi.

## RESULTS

### Construction of a phylogenetic tree and analysis of protein motifs.

**FIG 1:**
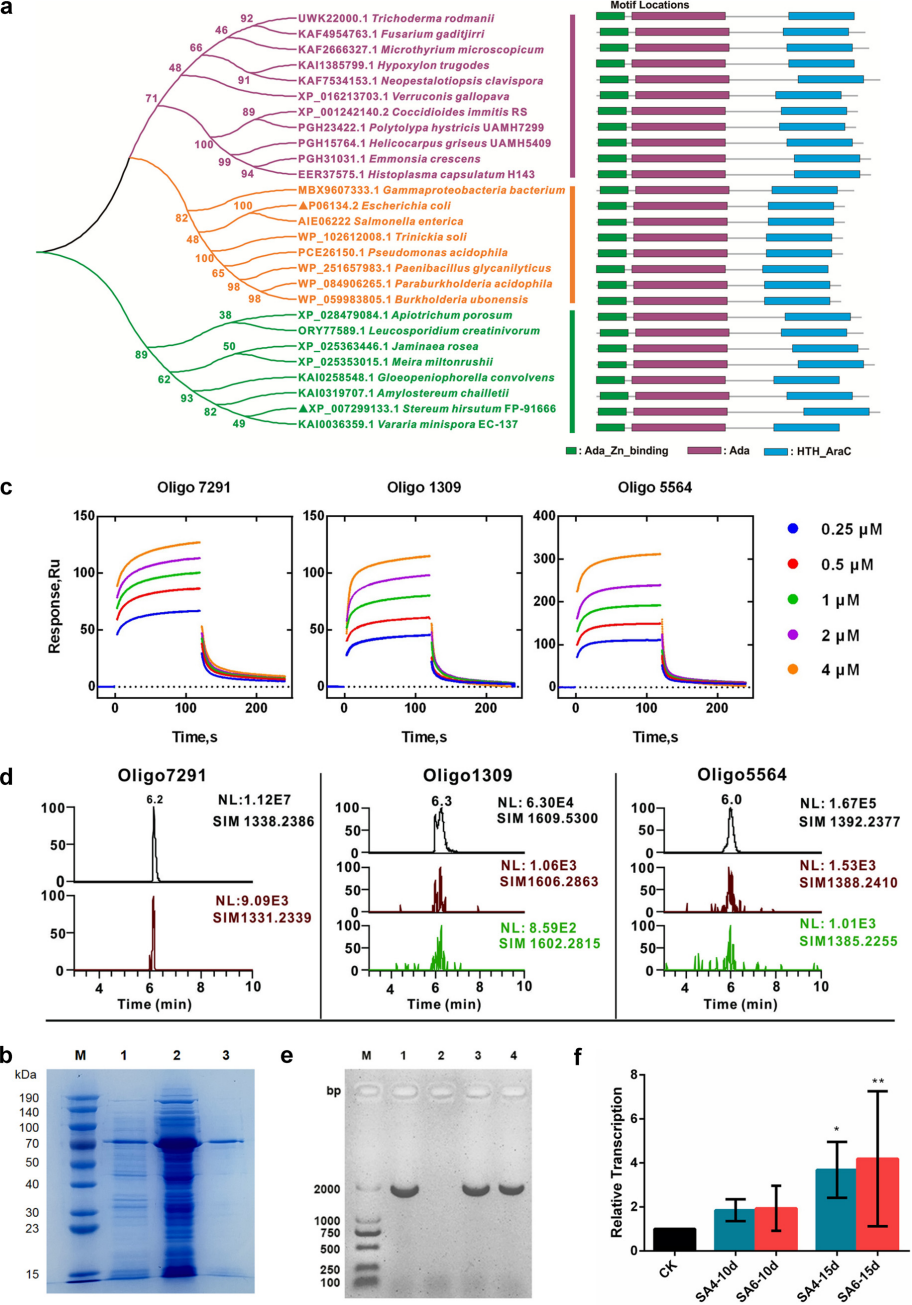
Functional characterization of SaraC. (a) Phylogenetic analysis of the SaraC homologous proteins. A phylogenetic tree was generated using the maximum likelihood method in MEGA X. Bootstrap values based on 1000 replicates are shown at the branching points. (b) SDS-PAGE analysis of the heterologous expression and purification of SaraC. Lane M: protein molecular weight marker; lane 1: soluble protein; lane 2: cell extract of total protein after induction with 0.1 mM IPTG at 16°C for 28 h; lane 3: purified fusion SaraC. (c) SPR analysis of the interaction of oligonucleotide (Oligo1309, Oligo5564, and Oligo7291 as analytes) with SaraC protein (activated with S-adenosylmethionine, as a capture). Different-colored traces indicate increasing analyte concentrations (ranging from 0.25–4 μM). (d) Chromatogram profiles of subtracts and products of oligonucleotides. In Oligo7291 reaction system, SIM1338.2386 shows the specific ion of Oligo7291 SIM peak at *m/z* 1338.2 [M–2H]^2–^; SIM1331.2339 shows the specific ion of product SIM peak at *m/z* 1331.2339 [M–2H]^2–^. SIM1609.5300 and SIM1392.2377 are the chromatograms of the Oligo 1309 and Oligo 5564 substrates, respectively; and the below chromatograms are the profiles of the corresponding de-monomethyl and de-dimethyl products, respectively. (e) PCR confirmation of the 2 transformants. Lane M, DL2000 DNA marker; Lanes 1 and 2: plasmid and WT as positive and negative controls, respectively; Lanes 3 and 4: the two transformants SA4 and SA6, respectively. (f) Relative transcription levels of *SaraC* quantified using RT-qPCR at 10 and 15 days. CK (β-tubulin) was used as the standard for statistical analysis of the transcription level of *SaraC* in the transformants relative to that in the WT strain under a given condition; error bars indicate the standard deviations.

To determine the possible functions of SaraC, we searched the NCBI database, the BLASTn program, and did not find any highly similar nucleic acid sequences. We then performed BLASTp using the translated amino acid sequence and found several homologous sequences from basidiomycetes and a large number of homologous sequences from bacteria. The result showed that SaraC contained 3 conserved domains: AdaA, Ada_Zn_binding, and HTH_AraC. Using the NCBI Conserved Domain Search, we selected 27 sequences that contained those conserved domains but belonged to different genera. As shown in the unrooted tree, 3 distinct clades were apparent (Fig. 1a): basidiomycete clade, bacterial clade, and ascomycete clade. Surprisingly, bacterial and ascomycete clades are near neighbors. Among selected sequences, the structure and function of P06134.2 from Escherichia coli have been more thoroughly studied (18).

MEME online software was used to identify and analyze the conserved motifs of SaraC-like protein sequences. The results showed that there were 3 conserved motifs in SaraC-like protein (Fig. 1a): Motif 1 contains 21aa and belongs to metal binding domain of Ada; Motif 2, which contains 50 aa, belongs to the AdaA protein of methylphosphotriester-DNA–protein-cysteine methyltransferase; Motif 3 contains 39 aa and belongs to the HTH_AraC family. These motifs were related to certain functions of the SaraC-like protein. In E. coli, the motifs of Ada_Zn_binding (zinc finger-structure with methyltransferase) and Ada are conserved domains of Ada protein (18). Ada, a suicidal demethylated protein, activates by capturing the methyl group in the methyl-containing phosphotriester by the *N*-terminus and is demethylated for the purpose of DNA repair at the O^6^-^Me^G of C-terminus (18). In addition, Ada is also a transcriptional regulator in fungi, activating the corresponding gene by transferring the methyl of O^6^-^Me^G to the cysteine residue of the Ada protein (19, 20). The HTH_ARAC motif is the conserved domain of the AraC transcription regulator, which is operative in E. coli (21). Until now, the function of proteins in this category in all eukaryotes has not been reported, but the function and reaction mechanism in bacteria have been studied more clearly. According to protein structural domain prediction and analysis, SaraC protein has a DNA binding structural domain that recognizes and binds nucleic acid sequences, as well as an ARA_C structural domain, which functions as a transcriptional regulator in E. coli.

### Heterologous expression and assay of SaraC activity *in vitro*.

The His_6_-tagged SaraC enzyme was expressed in E. coli BL21(DE3), purified by Ni-NTA chromatography, and detected using SDS-PAGE. The apparent molecular weight of recombinant His_6_-SaraC protein, as judged by relative mobility on SDS gels, is slightly higher than the predicted weight based on the deduced amino acid sequence (54.9 kDa) (Fig. 1b), which is a consistent feature of EmBP-l likely to be caused by the strong basic charge of the DNA binding domain (22).

Here, we performed a fusion His_6_-tagged SaraC interaction with methylated DNA using surface plasmon resonance (SPR) spectroscopy. This technology is widely used to detect DNA/DNA, DNA/protein, and small molecule/protein interactions; the interaction between analytes was detected in real-time by recording changes in the refractive index of nano-gold film on the chip surface (23, 24). In our experiment, 3 oligonucleotides were used as the analytes, having lengths of approximately 9 to 21 bp and methyladenine or methylcytosine in the middle of the sequence: 6mA, CpG and CHH (Table S1). The kinetic curves representing SaraC interaction with these 3 oligonucleotides were fitted using Biacore analysis software after deducting the reference channel and zero concentration (Fig. 1c). Interestingly, the SaraC protein exhibited different affinities when binding to the 3 oligonucleotides; the strongest affinity was observed for the SaraC-Oligo5564 and -Oligo7291 complex, which had *K*_D_ values of 8.208 × 10^−7^ and 6.624 × 10^−7^ M, and possessed the best response value. Furthermore, the length of the sequence and the number of methyladenines or methylcytosines did not appear to noticeably affect the protein binding. Oligo1309 has 2 methyladenines and the longest sequence length, but its affinity for SaraC was weak (1.672 × 10^−6^ M). This means that SaraC selectively prefers binding the C-site sequence. In summary, SaraC protein can both react with ^5m^C and ^6m^A oligonucleotides. Following the observation of methylated DNA binding to SaraC, we further analyzed the enzymatic products of these reactions using ultraperformance liquid chromatography mass spectrometry (UPLC-MS).

The demethylated products corresponding to oligo7291 exhibited the selected ion monitoring (SIM) peak at 6.00 min and was identified by its [M–2H]^2–^ ion at *m/z* 1331.5, while the peak substrate at 6.00 min corresponds to *m/z* 1338.2 [M–2H]^2–^. Similarly, in the electrospray ionization mass spectrometry (ESI-MS) spectrum of the sample incubated with oligo1309, two demethylated products were detected: one product corresponding to the removal of one methyl was located at *m/z* 1606 [M–4H]^4–^ and the other product of removing 2 methyl groups appeared at *m/z* 1602 [M–4H]^4–^ (Fig. 1d). Moreover, the corresponding demethylated products of oligo5564 were observed at *m/z* 1388 [M–4H]^4–^ (removal of 1 methyl group) and 1385 [M–4H]^4–^ (removal of 2 methyl groups), respectively (Fig. 1d). Our results showed that the abundance of the demethylated products was very low. The molecular weight of oligonucleotides is large, and there is a multi-charged quasi-molecular-ion peak while the demethylation by SaraC protein is also a suicidal process; consequently, methyl group is removed from substrate, and the corresponding molecular protein is inactivated (25).

### *SaraC* overexpressed in *S. hirsutum*.

By using an Agrobacterium tumefaciens–mediated genetic transformation approach, 2 over-expressed transformants were obtained, named SA4 and SA6. These 2 transformants were selected by hygromycin and confirmed by PCR using the total genomic DNA as a template (Fig. 1e). On the electrophoretic gel, SA4 and SA6 showed the expected 1949 bp product, whereas WT yielded negative results (Fig. 1e). Transcriptional analysis of *SaraC* via quantitative reverse transcription-PCR (qRT-PCR) demonstrated that expression of *SaraC* was upregulated in the 2 transformants compared to that detected in the WT strain. Specifically, the *SaraC* mRNA levels in SA4 increased to 185.3% and 368.7% at days 10 and 15, respectively, and those of SA6 to 193.9% and 419.0% at days 10 and 15, respectively (Fig. 1f). These results showed that the *SaraC* expression was successfully enhanced by A. tumefaciens–mediated random insertion of overexpressed *gpdA-SaraC*. Owing to the A. tumefaciens–mediated genetic transformation, *SaraC* was inserted into different locations in the genome of SA4 and SA6. We compared the difference in growth rate between WT and OES transformants on potato dextrose agar (PDA) and modified corn meal agar (CMA) solid media: With faster 7-day growth rate, SA6 obviously grows more slowly than SA4 and WT (Fig. S1).

### Comparative analysis of genome-wide DNA methylation levels in WT and OES transformants.

To explore the variation in DNA methylation of 2 OES transformants and WT, we conducted a whole-genome DNA methylation analysis of 2 OES transformants and WT using Nanopore Technologies (Table S2). These reads were mapped to the S. hirsutum genome (GenBank assembly accession: GCF_000264905.1) at mapping rates of 94.1–96.1%, corresponding to an average of 1,218,800 mapped reads for each sample. The genome-wide methylation level data showed that the methylation rates of CpG and 6mA were very different, while those of CHG and CHH did not appear to differ between the WT and OES transformants (Table S3). The methylation rates were as follows: 14.42% and 16.34% for CpG and 6mA in SA4-10, respectively; 14.29% and 15.69% for CpG and 6mA in SA4-15, respectively; 12.00% and 15.87% for CpG and 6mA in SA6-10, respectively; and 11.94% and 13.57% for CpG and 6mA in SA6-15, respectively. In comparison, the CpG and 6mA methylation rates for the WT were 15.23% and 13.74% at 10 days, respectively, and 15.15% and 13.62% at 15 days, respectively. Obviously, the methylation level of CpG sites in the OES transformants was lower than that of the WT, while the 6mA methylation levels of the transformants were higher than that of the WT. The main reason was that the 6mA sites (Table S4) meeting sequencing depth in the transformants were more than those of the WT according to the algorithm.

**FIG 2:**
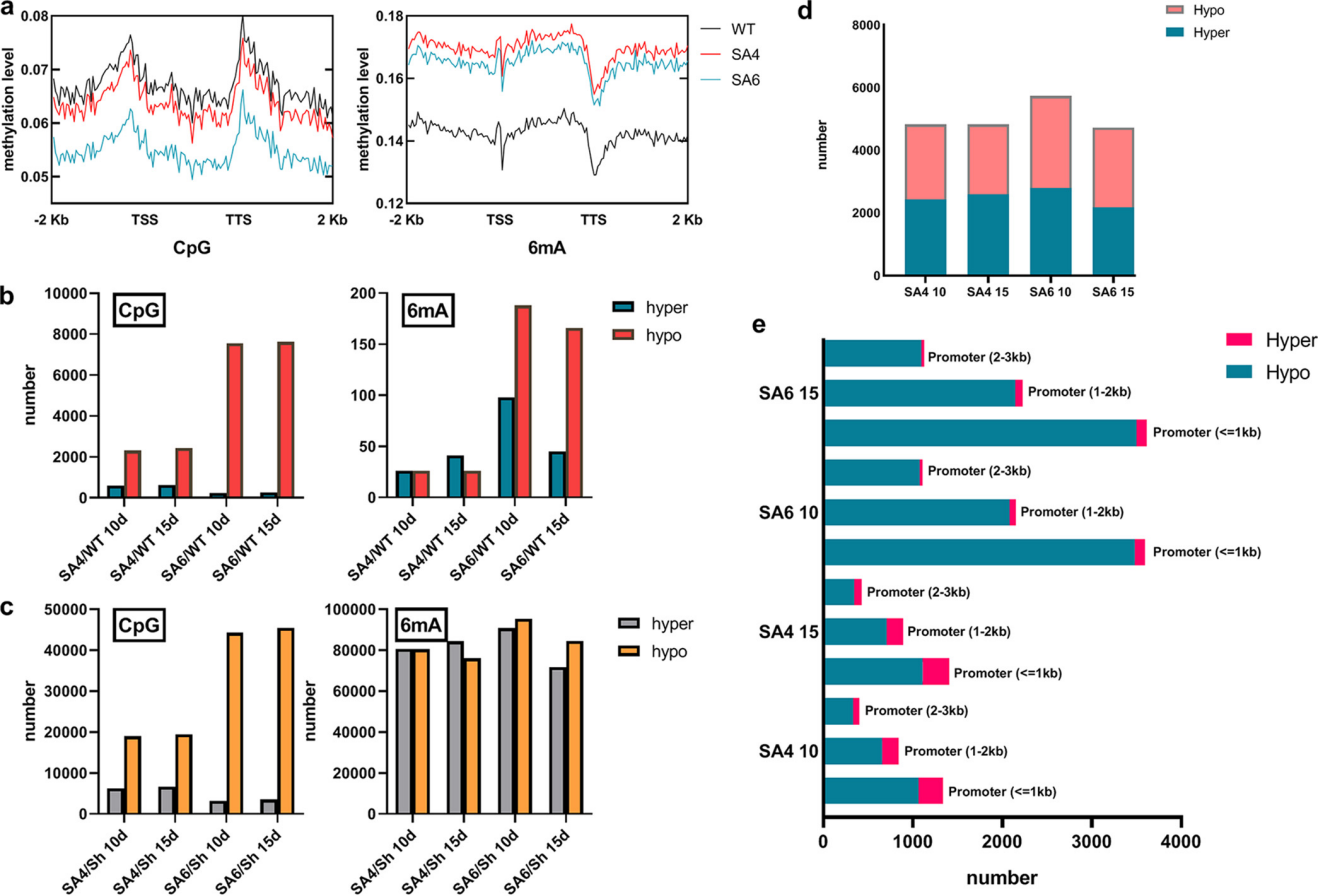
Methylation level of SA4 and SA6 compared with the WT. (a) The CpG and 6mA methylation level of TSS. (b) Number of different methylation regions (DMR) in CpG and 6mA; hyper and hypo represent significantly higher and lower methylation, respectively, in SA4 and SA6 samples relative to the control. (c) Number of different methylation loci (DML) in CpG and 6mA. (d) The number of hyper and hypo 6mA-DMLs in the TSS downstream (100 bp). (e) The number of hyper and hypo CpG-DMRs in the promoter area.

In different regions of the genome, the efficiency of methylation varied significantly. As known, the methylation loci near the transcription start site (TSS) will affect the gene expression. Therefore, we counted the average methylation level of genome, upstream promoter (2 kb upstream of TSS), and downstream region (2 kb downstream of TSS) by dividing each region into 50 bins and counting the methylation level of each bin. The average methylation level of TSS is lower than the WT in CpG, which possibly results in an increase in gene expression (Fig. 2a). Furthermore, CpG island (CGI) is a low methylation level region rich in CpG dinucleotides. CGI usually exists in 5’UTR and promoter areas and maintains a low methylation level in order to promote gene expression (26, 27). We found that the 2 OES strains of CGI remain at a lower methylation level than that of WT (Fig. S2). SaraC likely stabilizes the CGI at a low methylation level. Interestingly, the situation is on the contrary in 6mA, the 2 OES strains’ methylation level is higher than the WT’s, which can also impact the transcription because 6mA will mark the TSS and facilitate DNA strand separation (Fig. 2a) (28).

The 5mC always impact the transcription when they clump together in an area, so that the different methylation regions (DMR) do indicate biology credibility as known. Further, in 6mA, some reports suggested that the level of expression is regulated independently, so we focus on the different methylation loci (DML). The 2 OES transformants were compared with the WT to analyze the different methylation loci (DML) and the different methylation regions (DMR), and different standards were used by analyzing the 4 methylation loci, of which 6mA was based on *P < *0.05 and methylation specificity (MS) > 0.2; for the other 3 loci (i.e., CpG/CHG/CHH), the same *P* value and MS were used (i.e., *P < *0.01 and MS > 0.5, respectively). Interestingly, in our study, SA4 and SA6 showed different methylation levels but the same tendency among the 4 methylation loci, as well as different transcription levels of *SaraC* (Fig. 1f). They both also have more hypomethylation DMR number than hyper in CpG and 6mA (Fig. 2b), and almost same number of hypo and hyper DML in 6mA (Fig. 2c).

In addition, we analyzed the CpG-DMR distribution area of the promoters, which divided the promoters into 3 categories: promoters less than 1 kb, between 1 and 2 kb, and between 2 and 3 kb for CpG-DMR. The 6mA-DML in the TSS, downstream (100 bp) was also analyzed (29, 30). The result showed that the 6mA in downstream 100 bp around TSS is in a large proportion, there is not much difference with the number of the hyper and hypo, which still can probably result in gene expressing because the 6mA work independently (Fig. 2d) (30). The CpG also showed the strong hypomethylation in the promoter area, especially in the area less than 1 kb (Fig. 2e). All methylation phenomena indicate that SaraC does affect transcription.

**FIG 3:**
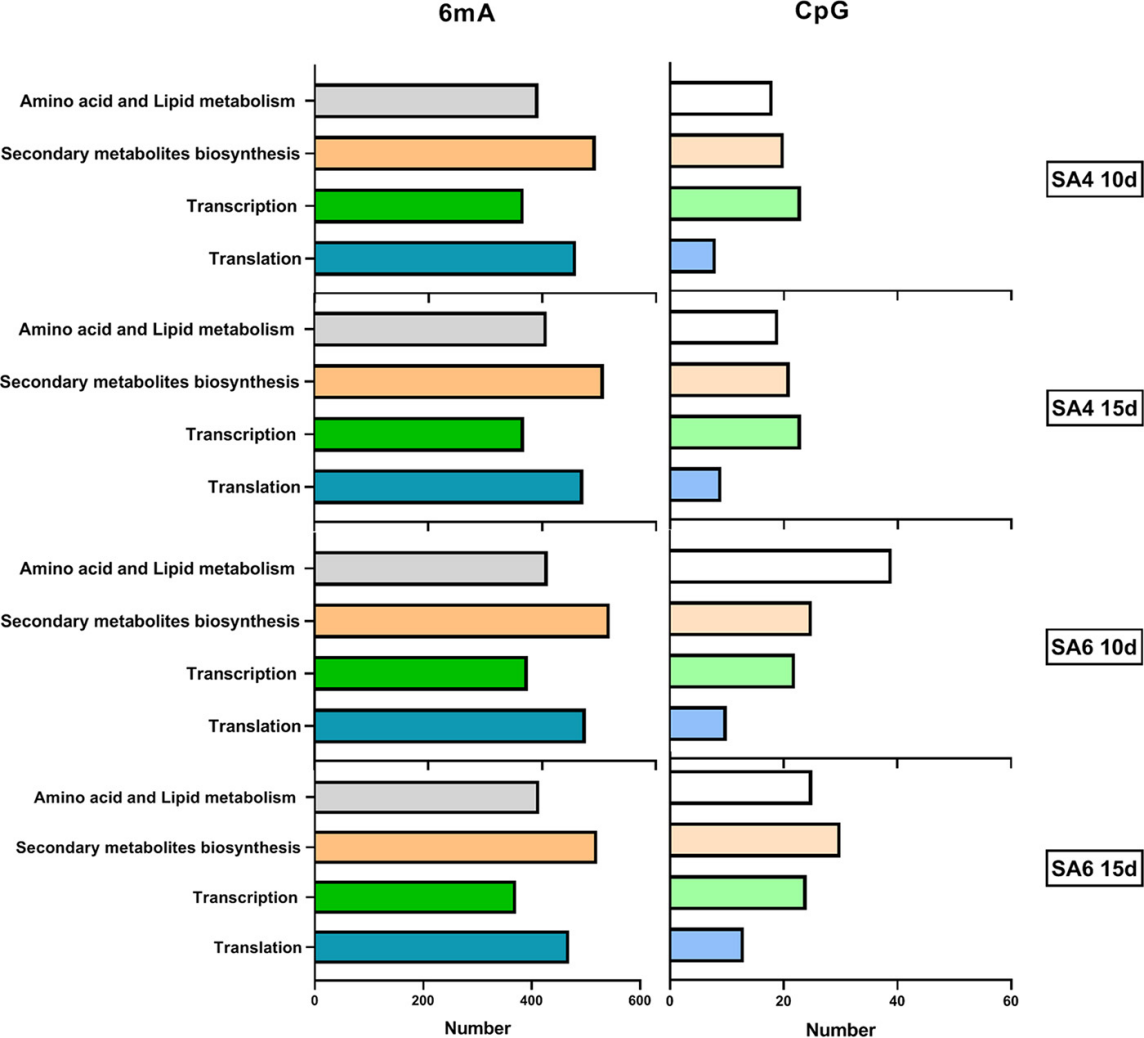
The genes associated 6mA-DML and CpG-DMR analysis of different methylation-region-related genes of CpG by GO/COG/KOG, and separate into 4 categories include amino acid and lipid metabolism, secondary metabolism, transcription, and translation.

To analyze the gene functions associated with 6mA-DML and CpG-DMR, we annotated and classified them using normal databases, including GO, COG, and KOG (Table S5). Among these annotated genes, we focused on those related to 4 types of pathways: amino acid and lipid metabolism, secondary metabolism, transcription, and translation. Surprisingly, the gene associated 6mA-DML has a large number of secondary metabolism related genes, and translation related genes (Fig. 3). These data prompt that the 2 OES strains may be in high metabolism, especially in secondary metabolism.

### Transcriptomes analysis of WT and OES transformants.

**FIG 4:**
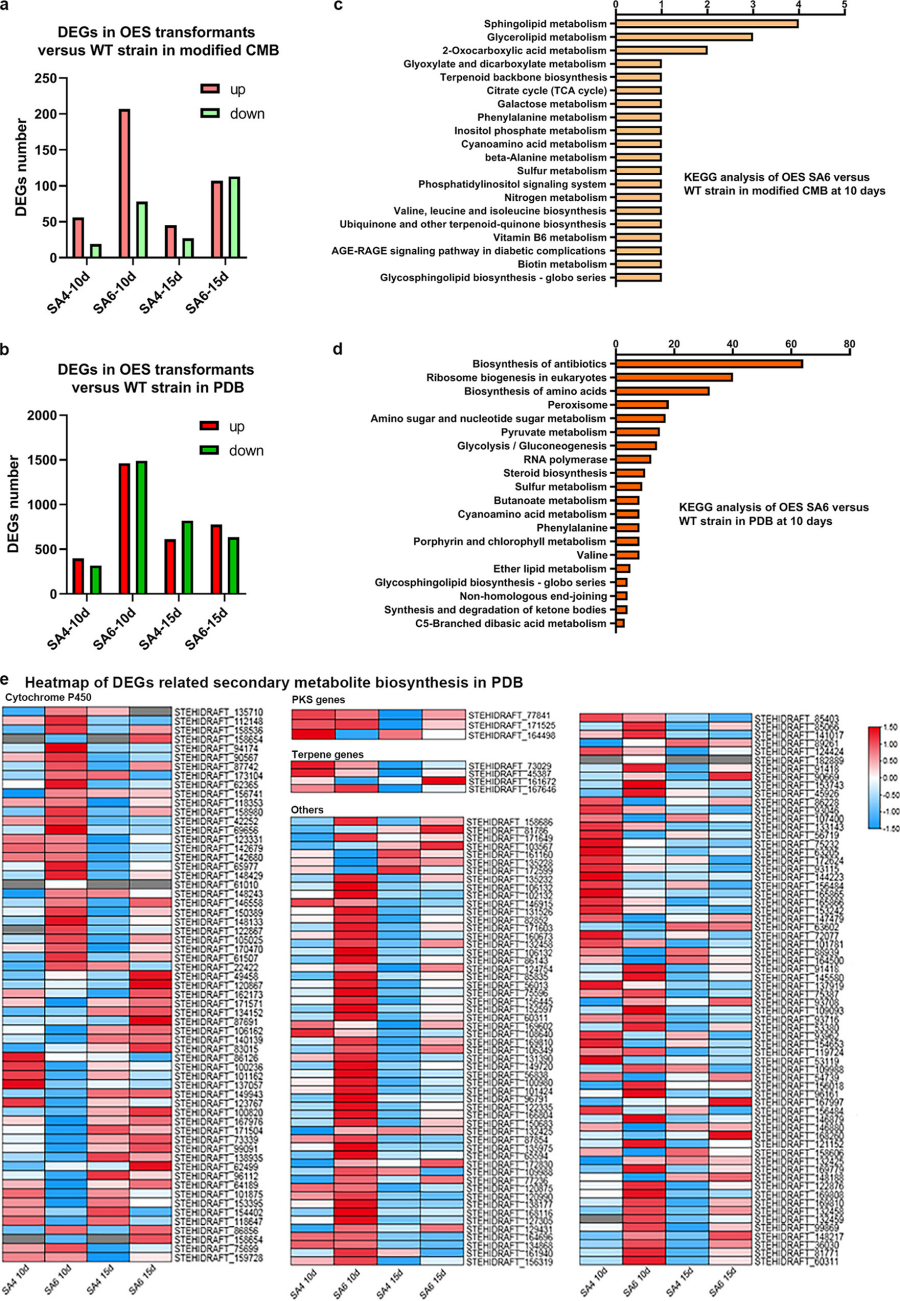
Comparison of DEGs between WT and overexpress SaraC strains. (a) The number of upregulated and downregulated DEGs in the OES strains versus WT strain in modified CMB. (b) The number of upregulated and downregulated DEGs in the OES strains versus WT strain in PDB. (c) KEGG enrichment analysis of DEGs in the OES strains versus the WT strain at 10 days in modified CMB. (d) KEGG enrichment analysis of DEGs in the OES strains versus the WT strain at 10 days in PDB. (e) Heatmap of other secondary metabolite biosynthesis genes.

As known, 6mA is strongly associated with gene expression (30). When it enriches specially around TSS, it’s expected to facilitate DNA strand separation and control the transcription, like a mark. Also, the quantity of 6mA on genes did not have any clear impact on expression level, meaning, while 6mA acts to facilitate transcription, the actual level of expression is regulated independently. To determine the expression differences between WT and OES transformants, we performed RNA sequencing experiments on WT, SA4, and SA6. A total of 652 genes were significant at this level, using a Bonferroni threshold based on *P < *0.05. Among these genes, we retrieved those showing a significant differential expression in comparison with WT from the same cultural periods (Fig. 4a). The KEGG enrichment analysis showed differentially expressed genes (DEGs), but there are only 2 secondary metabolic pathways: terpenoid backbone biosynthesis and other terpenoid-quinone biosynthesis (Fig. 4c). Based on this result, we further found a sesquiterpene biosynthetic gene cluster (named as Cluster 2.4), in which the expression levels of most genes are significantly upregulated (Table S6). Interestingly, STEHIDRAFT_50014, STEHIDRAFT_118347, STEHIDRAFT_91274 and STEHIDRAFT_107839 are in hyper methylation level of 6mA around TSS, and also highly expressed. The rest genes in Cluster 2.4 are also highly expressed as Table S6 shows.

Additionally, to see whether the methylation function of SaraC is stable in different cultures, we checked the expression differences in PDB (Fig. 4b and Table S7), and there are more secondary metabolite related genes highly expressed than oligotrophic conditions (modified corn meal broth [CMB]). We categorized DEGs belonging to secondary metabolite biosynthesis clusters, typically in the polyketide and terpene synthase gene clusters, with the differential genes from the former accounting for 6.5% of the total, and some of them being significantly upregulated at 10 days in the SA6 strain (Fig. 4d). The terpene synthesis gene cluster accounted for 13.1% of the total, and the majority of its genes showed upregulation at 10 days. Additionally, other relative genes of secondary metabolites were identified (Fig. 4e). In the biosynthesis of secondary metabolites, basic skeletons (e.g., terpenes, polyketides, and non-ribosomal peptides) were synthesized, and then a series of compounds were produced by a large number of post-modifications such as hydroxylation, phosphorylation, glycosylation, prenylation, as well as oxidation–reduction reaction. These post-modifications not only increase the structural diversities, but the partial redox reaction causes the rearrangement of the carbon skeleton to produce a new skeleton; finally, a large number of secondary products with various physiological activities are produced (31). The cytochrome P450 family is considered a key type of enzymes for the biosynthesis of secondary metabolites and their derivatives owing to the wide variety of reactions they catalyze, and the diversity of their reactive substrates (32, 33). Therefore, we annotated and classified the DEGs associated with secondary metabolism that appeared in the transcriptome and found that genes belonging to P450 accounted for 19% of the total DEGs, and most of them were significantly upregulated at 10 days in the SA6 strain (Fig. 4e). Based on this, a variety of secondary metabolites may be detected and isolated in OES transformants.

### Metabolome differences among WT and OES transformants.

**FIG 5:**
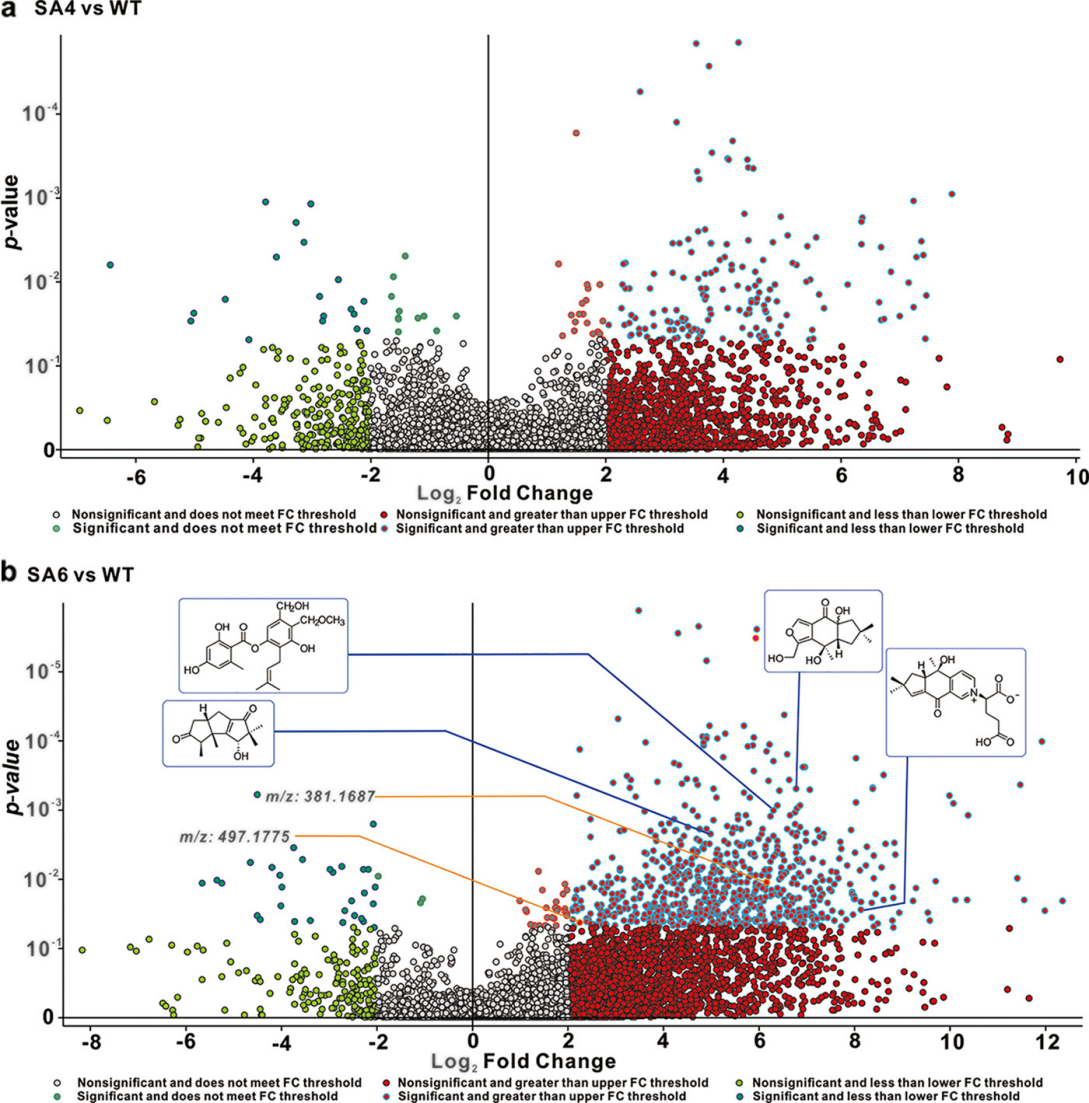
Volcano plot representation of global changes in the *S. hirsutum* metabolome in response to overexpression of *SaraC.* Significance cutoffs were *P = *0.05 (Bayes moderated *t*-tests) and FC = 2. Each dot represents an individual compound (within ±10 ppm in mass), and the probability of that quantitative observation being statistically significant is indicated by a *P* value on the *y* axis (determined using the standard linear model within SIEVE software). (a) Volcano plots of WT versus SA4. The 157 compounds on the right half of the plot are present at significantly higher levels in the SA4 samples. The 19 compounds on the left-hand portion of the diagram are present at significantly higher levels in the WT samples. (b) Volcano plots of WT versus SA6. The 755 compounds on the right half of the plot are present at significantly higher levels in the SA6 samples. The 31 compounds on the left-hand portion of the diagram are present at significantly higher levels in the WT samples. Displayed in the insets are the responses of several known metabolites.

We used untargeted metabolomics to evaluate the function of SaraC to activate secondary metabolites in the basidiomycete S. hirsutum. Combining all the analyzed strains, 8,972 unique molecular species were detected using UPLC-HR-ESI-MS. The high-resolution mass spectrometric signals from different isotopes and adduct peaks were combined so that the vast majority of molecular species represented individual metabolites produced by the corresponding strain. We aimed to determine the secondary metabolite differences in WT and 2 OES transformants, and displayed the data as a volcano plot for visualization. Using significance cutoffs of a FDR adjusted *P* value (<0.05) and a fold change difference of >2, we observed that 157 metabolites were upregulated in SA4 versus WT, while 19 metabolites were downregulated in SA4 versus WT (Fig. 5a); furthermore, 755 metabolites were upregulated in SA6 versus WT; while 32 were downregulated (Fig. 5b). In order to determine the upregulated compound structures, we assembled a structural library containing 107 metabolites from S. hirsutum after removing common steroids and fatty acids (34–36).

The complete masses of upregulated compounds are used to search the structural library and other libraries (MZCloud, ChemSpider, and MZvault). When searching these databases with a mass tolerance of 10 ppm, 41 annotations were verified and displayed in Table S8. Annotated MS/MS spectra (Fig. S3 to S19) were used to further verify the ability of tandem MS and manual interpretation to identify known compounds. The 41 compounds mainly included 3 categories: sesquiterpenoids (structural classifications as hirsutanes, illudalanes, and drimanes), simple aromatics, and polyketide hybrids (Table S8). Subsequently, we quantitatively analyzed these and other metabolites in response to 2 OES transformants. Over-expression of *SaraC* led to a 4-fold increase in metabolite production of >97% of known compounds in OES SA6 compared to WT (Table S8); a greater than 10-fold change was observed for >88% of known metabolites relative to WT. Although the relative contents of these known compounds in SA6 and SA4 are significantly upregulated, the degrees of upregulation in SA6 were significantly higher than those in SA4 (Table S9). Furthermore, there are also a large number of metabolites with undefined structures that are significantly upregulated in SA6. In addition to the compounds identified by the metabolome, there are several molecular ion peaks that are unknown. Although the database was searched, some compounds were identified as chalcone-type compounds, such as brosimacutin C (*m/z*: 343.1510 [M+H]^+^; RT: 17.44 min), elaeocyanidin (*m/z*: 361.1276 [M+H]^+^; RT: 13.437) and rubone (*m/z*: 375.1428 [M+H]^+^; RT: 16.995) (Table S10). However, there is no PKS type III biosynthetic gene cluster in the S. hirsutum genome (37). Based on the predicted structural characteristics and the actual characteristics of the identified compounds, it is believed that these compounds are most likely MS-3 and sterenin H–type compounds: the hydroxyl group of phenolic compounds is connected to isopentenyl and orsellinic acid, forming a hybrid compound with a unique structure (38). For visual comparison, we selected several unknown structures that were significantly upregulated when comparing the 2 groups (Fig. 5b). In the next experiment, we will ferment OES transformant SA6 in order to obtain those metabolites.

### Novel metabolites from OES transformant SA6.

The crude extract (6.6 g) resulting from the fermentation of OES SA6 transformants was isolated and purified guiding by LC-MS, which helped us eliminate the repeated separation of known compounds to finally obtain 2 target compounds.

**FIG 6:**
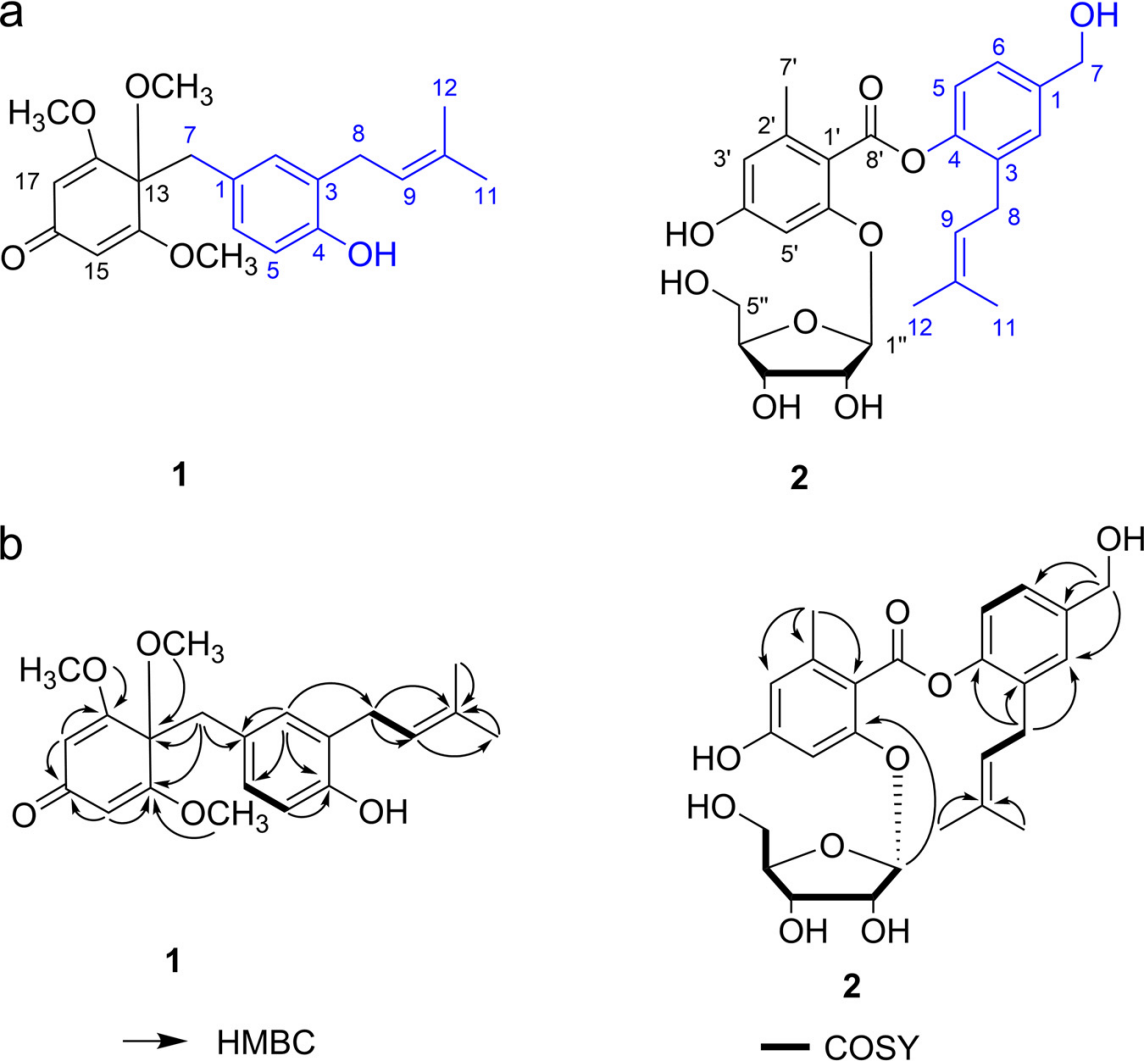
Identification of metabolites. (a) Novel structures from SA6. (b) Selected HMBC (arrows) and ^1^H–^1^H COSY (bold bond) correlations of new structures.

Compound 1 was isolated as a colorless, amorphous solid. Its molecular formula C_21_H_26_O_5_Na was determined using HR-ESI-MS (*m/z* 381.16700 [M + Na]^+^, calculated as 381.16697). The ^1^H NMR spectrum exhibited signals for 3 methyl groups at *δ*_H_ 1.73 (3H, s), 1.77 (3H, s), 3.15 (3H, s), and 3.80 (6H, s × 2), and 4 olefinic protons at *δ*_H_ 5.43 (2H, s × 2), 5.24 (1H, d, *J *= 7.3 Hz), 6.63 (1H, s), 6.58 (1H, d, *J *= 8.7 Hz), and 6.64 (1H, d, *J *= 8.7 Hz). The ^13^C NMR, DEPT, and HSQC spectra of compound 1 revealed 21 carbon signals that represent 5 methyl groups, 2 methylene groups, 6 methine groups, and 8 quaternary carbons (Table S11). A degree of unsaturation analysis of the ^1^H and ^13^C NMR data implies that compound 1 contains one isopentenyl unit (*δ*_C_ 29.2, 121.6, 134.6, 17.8, and 25.8) and one cyclohexa-2,5-dienone unit (*δ*_C_ 80.4, 168.4 × 2, 104.9 × 2, and 186.9 ppm) (Fig. 6a) (39). The detailed structure was determined using the COSY and the HMBC correlations. In the COSY spectrum, 2 fragments were deduced by the clear correlations of H-5/H-6 and H-8/H-9 (Fig. 6b), respectively. The HMBC (Fig. 6b) data indicated the presence of 3,4,5-trimethoxycyclohexa-2,5-dien-1-one moieties by correlations between H-15/17 protons (*δ*_H_ 5.43) and C-13, C-14/18, and C-16; between OMe-14/18 (*δ*_H_ 3.80) and C-14/18; and between OMe-13(*δ*_H_ 3.80) and C-13. In addition, correlations existed for 2 Me protons (*δ*_H_ 1.73 and 1.77) with C-9 and C-10, for H-2 (*δ*_H_ 6.63) with C-4 and C-1, and for H-6 (*δ*_H_ 6.64) with C-4 and C-2, establishing the presence of 2-pentenyl-phenol moieties. The junctions of the 2 moieties were connected by a methylene group, as deduced from the following HMBC correlations (Fig. 6b): H-7 (*δ*_H_ 3.22) with C-13 and C-1 with C-14/18. Thus, after piecing together the correlations, the structure of compound 1 was determined as a rare hybrid polyketide. Based on the structural characteristics of compound 1, it appears to be a metabolite synthesized through a type III PKS pathway. However, there is no PKS3 biosynthetic gene cluster in S. hirsutum genome (37), so we speculated that compound 1 was a hybrid polyketide derived from the hydroxyl group of phenolic compounds and connected to isopentenyl and orsellinic acid. According to the above data, the structure of compound 1 was elucidated as in Fig. 6 and named to be sterenin N.

Compound 2 was obtained as a colorless, amorphous solid. Its molecular formula C_25_H_30_O_9_Na was determined using HR-ESI-MS (497.17749 [M + Na]^+^, calculated as 497.17820). The ^13^C NMR, DEPT, and HSQC spectra of compound 2 revealed 25 carbon signals that represent 3 methyl groups, 3 methylene groups, 10 methines (including 6 olefinic methines), and 9 quaternary carbons (Table S11). Based on spectroscopic data, compound 2 was a hybrid of the following groups: ribose; orsellinie acid; and 4-hydroxy-3-(3-methylbut-2-en-1-yl)-benzene-methanol (Fig. 6a) (38, 40). The 2D-NMR experiment (Fig. 6b) showed several key correlations: the protons of 2 methyl groups (*δ*_H_ 1.53 and 1.62) correlated with C-9 and C-10; H-8 (*δ*_H_ 3.29) of the methylene group correlated with C-9, C-3, and C-4; H-7 (*δ*_H_ 4.61) of the methylene group correlated with C-6, C-2, and C-1; H-9′ (*δ*_H_ 2.62) of the methyl group correlated with C-1′, C-5′, and C-6′; and H-1′′ (*δ*_H_ 5.71) of the methine group correlated with C-2′. The small coupling constant of the anomeric proton (4.4 Hz) indicated an α-glycosidic linkage (Fig. 6b). The pentose was assigned as α-d-ribofuranose by comparison of the spectral data with that reported in the literature (41). The structure of compound 2 was shown in Fig. 6 and named as sterenin O.

The activities of new compounds were not tested due to sample limitations. According to the structure type of the identified compounds, we speculate that they maybe possess the potential corresponding activities. For example, tarennane and tarennone have two very similar structures, but only tarennane has antioxidant activity of protecting against H_2_O_2_-induced PC2 cell injury, of which tarennane has a unique structural unit of 3,4,5-trimethooxycyclhexa 2,5-dienone (39), and compound 1 also has the same structural unit (Fig. 6). Also, as compound 2 is an analog of MS-3, its’ structure contains a five-membered ribose group (Fig. 6), which may show α-glucosidase inhibition (42).

## DISCUSSION

6mA, which mainly exists in prokaryotes, relates to DNA replication, regulation of gene expression, and cell defense (43, 44). Nowadays, studies prove that 6mA is an important methylation mechanism in eukaryotes, both in regulating nucleosome positioning and affecting chromatin organization, inhibiting DNA replication, and activating transcription (45). There are still so many mysteries, such as how it works and how it impacts transcription that still have no clear conclusions. Ten-eleven translocation (TET) dioxygenase is the only known DNA demethylases that works on dsDNA (46), and one of its homolog enzyme CMD1 from the fungus Coprinopsis cinerea shows high abundances in 6mA modification by oxidizing 6mA to the intermediate N6-hydroxymethyladenosine (47). Our results show that SaraC functions in both 6mA and 5mC modifications, which removes the methyl directly based on the *in vitro* enzyme activity (Fig. 1). Concurrently, the overexpression of SaraC stimulates significant production of metabolites. We detected more 6mA in OES’s genome than WT’s, and the hyper 6mA-DML’ number is more than the hypo’s, meaning that SaraC activates gene expression through the 6mA modification (Fig. 2 and Table S3 to S5). Furthermore, the SaraC also has the demethylation function in 5mC, the hypomethylation DMR of CpG/CHG/CHH are higher than the hyper, especially in CpG. The number is also larger in the promoter area, which is 1 kb near TSS, so it can be considered to have the function of activating the gene expression. However, how it works to insert into adenosine and remove a methyl from cytosine is still unclear.

As the main modification mechanism in epigenetics (48), DNA methylation widely occurs in eukaryotes and plays an important regulatory role in eukaryotic growth and development, cell differentiation, and physiological metabolism (49). DNA demethylation has been shown to be successful in inducing the expression of secondary metabolic biosynthetic genes (50). According to this strategy, SaraC significantly reduces the CpG methylation level in the gene transcription region and increase the 6mA methylation level around TSS, causing most of the fungal gene transcription activity enhancement. This leads to the expression of previously cryptic gene clusters and the production of related natural products. This model is supported by the whole gene methylome, transcriptome, and metabolome, and validated our prediction by combing omics data through enhancing the structural identification of new compounds that express metabolites of transformants. Interestingly, the largest change in the expression of *SaraC* in this experiment greatly increased the abundance and diversity of secondary metabolites. This study illustrates the importance of selecting genes with adaA protein regulator as targets; they have been studied systematically in bacteria, but we speculate and confirm, for the first time, that it can globally regulate the biosynthesis of secondary metabolites in fungi. Such regulatory factors are ubiquitous in fungi, yet researchers have devoted more attention to their ability to repair DNA damage, ignoring their ability to biosynthesize in secondary metabolism. Our research will provide a new tool for the excavation and regulation of metabolites in fungi.

## MATERIALS AND METHODS

### Fungal strains, plasmids, and culture conditions.

The strain S. hirsutum FP-91666 was obtained as a gift from culture collection manager, Ms Rita Rentmeester from the Center for Forest Mycology Research, Northern Research Station, Madison, WI, USA. S. hirsutum FP-91666, S. hirsutum SA4, and SA6 were stored in the State Key Laboratory for Conservation and Utilization of Bio Resources in Yunnan, Yunnan University, China. Agrobacterium-competent cell lines AGL1 and Escherichia coli BL21(DE3) competent cells were obtained from Biomed. Plasmids pC-HYG-YR and pBARGPE1-Hygro-EGFP were obtained from MiaoLingPlasmid (Wuhan Miaoling Biotechnol Co.).

### Phylogenetic analysis of SaraC-like proteins and analysis of protein domains.

The genetic context of *SaraC* (GenBank: XM_007299071.1) and gene coding for Ada and HTH_AraC domains in mushroom genomes were comparatively investigated in NCBI. SaraC homologues were predicted by using BLASTp against protein sequences from fungi and bacteria. Considering the respective prototypes of SaraC-like proteins, the homology search identified 27 homologs of SaraC-like enzymes, which consist of Ada and HTH_AraC domains. Due to large differences in the domain location in the selected proteins, as well as their differences in length, the protein sequences were trimmed, and only the amino acid sequences of 3 motifs were retained. Finally, the phylogenetic relationships between SaraC and its homologs (containing Ada and HTH_AraC domains) were calculated using MEGAX software by applying the Maximum Likelihood statistical method, the Bootstrap method considering 1000 replicates as the phylogeny test, and the LG + G + I model. MEME online software (https://meme-suite.org/meme/tools/meme) was used to analyze the motif of these family members.

### Expression and purification of *SaraC*.

The *SaraC* CDS sequence was synthesized by the Beijing Genomics Institute (BGI, Beijing) in pET32, and transferred into E. coli BL21(DE3). The expression strains were cultured in 50 mL-Luria–Bertani (LB) broth supplemented with 50 μg/mL ampicillin at 37°C, shaken at 180 rpm overnight, and then transferred into a new 50 mL LB broth (in the proportion of 1:100), and cultured at 37°C for 1 h. Then, after adding 50 μL IPTG (100 mM) and ZnCl_2_ (100 mM), the strains were incubated for another 28 h at 16°C with shaking at 90 rpm. The induced E. coli BL21(DE3) cells were centrifuged at 5000 rpm for 10 min at 4°C to produce sediment. The sediment was suspended with ddH_2_O and centrifuged at 5000 rpm for 10 min at 4°C, re-suspended with Binding Buffer (20 mM Tris-HCl of pH 8.0, 100 mM NaCl, and 5 mM imidazole), and then sonicated on ice in 6 s periods at 6 s intervals for a total of 6 min. Finally, the mixture was centrifuged at 5000 rpm 4°C for 20 min. The resulting supernatant was mixed with Ni-NTA on ice for 1 h, and then poured into a column. When the liquid ran out, 10 mL Washing Buffer (20 mM Tris-HCl of pH 8.0, 100 mM NaCl, and 50 mM imidazole) was added 5 times, and the solution was transferred to a new bottle along with 10 mL Dute Buffer (20 mM Tris-HCl of pH 8.0, 100 mM NaCl, and 200 mM imidazole) to obtain fusion SaraC protein. A new ultra-centrifugal filter (30 kDa MWCO, Millipore) was doused with ddH_2_O, and 10 mL of the protein sample was added and centrifuged at 2000g for 20 min. Then, 5 mL of desalination buffer (50 mM Tris-HCl of pH 8.0 and 5 mL glycerin) was added and centrifuged again to concentrate the protein. The entire process was carried out at 4°C.

### Surface plasmon resonance measurements.

Surface plasmon resonance (SPR) binding affinities were performed on a Biacore T200 instrument (GE Healthcare) according to the protocols provided by the manufacturer and described in the previous reports (51, 52). The anti-his antibody was covalently immobilized onto the dextran hydrogel of the GE Healthcare chip CM5 by using the Anti-his Kit (GE Healthcare), both in the reference and experimental channels. The purified fusion His_6_-tagged SaraC was mixed with *S*-adenosylmethionine (SAM, 16 mM) and 1×PBS buffer (diluented 10xPBS buffer from GE Healthcare with 5% DMSO added, and pH adjusted to 7.5), and incubated at 37°C for 1 h. The mixture was flowed into the experimental channel (1200 s, flow rate of 10 μL/min, three times) and captured. The oligonucleotide samples (Oligo1309, Oligo5564, and Oligo7291) were synthesized by BGI (sequences available in Table S1), dissolved using 1× PBS buffer, and diluted 2-fold into different concentrations ranging from 250–8000 μM, while 2 controls (0 μM) were set at the beginning and end of the procedure. The samples were flowed into the reference and experimental channels; the association and dissociation times were both 120 s, and the flow rate was 5 μL/min. All measurements were carried out at 25°C and controlled using BIACORE Control Software. The association (K_a_) and dissociation (K_d_) kinetic constants were calculated using BIAevaluation 3.1 software with a simple 1:1 binding model. The equilibrium dissociation constant (*K*_D_) values were calculated according to *K*_D_ = K_d_/K_a_.

### Assay of SaraC activity *in vitro* and analysis of demethylated product using UPLC-ESI-MS.

As the substrate, the ^5m^C and ^6m^Aoligonucleotides were synthesized by BGI. The same sequence without ^5m^C and ^6m^A was synthesized by TsingKe Biological Technology as the product positive control. *S*-adenosylmethionine (SAM) acts as a methyl group supplier in the reaction. The reaction mixture included 10 μL oligo, 5 μL SAM, and 85 μL recombinant SaraC, which reacted at 37°C for 96 h.

The demethylated products of the oligonucleotide were detected using a Thermo Scientific Dionex Ultimate 3000 UHPLC system equipped with a Thermo high-resolution Q Exactive focus mass spectrometer (Thermo). The injection volume was 10 μL, and the chromatographic column was Hypersil Gold (50 mm × 2.1 mm, Thermo Fisher Scientific) with a particle size of 1.9 μm. The mobile phase was a gradient prepared from a DEAA solution (A) and methanol (B). The DEAA solution was prepared as follows: 1.49 mL of acetic acid was added to 400 mL deionized water, and then 3.6 mL of diethylamine was slowly added and the pH was carefully adjusted to 7.0 using acetic acid while mixing; water was then added to adjust the volume to 500 mL. Initially, 5% B was eluted for 2 to 15 min, and then the proportion of B was increased linearly to 95% at 9 min and held for 5 min; the proportion of B was then reduced to 5% at 16 min and held for 4 min, lasting 20 min. The flow rate of the mobile phase was maintained at 300 μL/min. Mass spectrometry was performed on a Thermo high-resolution Q Exactive focus system (Thermo Fisher Scientific), and the products were analyzed under negative-ion mode. The optimized conditions were as follows: sheath gas flow rate of 30 arbitrary units, auxiliary gas flow rate of 20 arbitrary units, spray voltage of -3.0 kV, and capillary temperature 200°C.

### Over-expressed *SaraC* in *S. hirsutum*.

To obtain the plasmid for *Agrobacterium*-mediated transformation that has a *gpdA* promoter, 2 plasmids, pC-HYG-YR and pBARGPE1-Hygro-EGFP, were digested by *Asis*I and *Bgl*II, dephosphorylated, and linked by T4 ligase (named pYUZ70). The *SaraC* gene fragment including 2 introns was cloned by the PCR of S. hirsutum. The primers used for PCR were as follows: pYUZ70 was digested by BamHI, and the fragment and lined vector were linked using In-Fusion. The fusion plasmid was named pYUZ71.

Mycelia of S. hirsutum were cultured in potato dextrose agar for 3 days, and the fresh mycelia were cut into small pieces and cultured in TG liquid medium (1% tryptone and 1% glucose) for 3 days. The strains were filtered and collected in bottles containing enzymatic hydrolysates (0.8 mg cellulase, 0.8 mg snailase, and 0.4 mg driselase in 10 mL MN buffer) for 6 h. Enzymatic hydrolysates were filtered, and protoplasts were collected and stored at 4°C for 2 h. The pYUZ71 plasmid was introduced into the AGL1 of A. tumefaciens according to previously described methods (53). A. tumefaciens strains of AGL1 harboring the binary vector pYUZ71 were grown to an optical density at 600 nm (OD_600_) of 0.5 to 0.6. This was achieved by using 50 mL LB broth prepared at 28°C and 180 rpm, which was supplemented with 50 μg/mL kanamycin. Induction medium (IM) was used for the transformation using A. tumefaciens. The bacterial solution was diluted in 50 mL of IM containing 0.3 mM acetosyringone (AS) to an OD_600_ of 0.15, and then grown in the dark at 28°C and 180 rpm to an OD_600_ of 0.5 to 0.6. The cells were harvested by centrifugation at 5000 rpm for 5 min. The induced A. tumefaciens solution was mixed with the prepared protoplast suspension in a ratio of 1:1, and then 100–150 μL of the mixed solution was coated on an IM solid plate in the dark at 22°C, followed by coculturing for 48 h or until the mycelium grew out. Two validation steps were performed in the screening of transformants. The first step was to cover the medium with a layer containing 500 μg/mL cefotaxime to inhibit the growth of A. tumefaciens. After the growth of the mycelium, a layer containing 150 μg/mL hygromycin B was added to cover the medium for the second screening, allowing transformants to grow on the resistance selection plate. Through PCR verification (ks-F: 5′-CGAGGTCGACGGTATCG-3′; ks-R: 5′-CGATACCGTCGACCTCG-3′), 2 overexpressed *SaraC* (OES) transformants were obtained and named S. hirsutum SA4 and SA6.

RNA was extracted from the mycelia (SA4, SA6, and WT) using a total RNA miniprep kit (Axygen) at 10 and 15 days. A TaKaRa reverse transcription kit (TaKaRa Biomedical Technology) was used to synthesize cDNA as the template for qPCR. Quantitative real-time PCR was performed using a SYBR green Master (Roche Applied Science) on a Roche LightCycler 480 system (Roche Applied Science). β-tubulin was used as an internal control. The primers used for PCR were as follows: β-tubulin, 5′-ACCGCGCAGTGTGACATCCC-3′ (forward), 5′-AGCGGTCGCATCTTGGTATT-3′ (reverse), *SaraC*, 5′-CCTGGAGGGGCATGTTGGAC-3′ (forward), and 5′-ACACCCTATCGCCTCTTCAT-3′ (reverse). The relative transcription level of each gene was calculated as the ratio of the transcription level in the OES transformants SA4 and SA6 to that in the WT strain at a given time point according to the 2^-ΔΔCT^ method. All assays were repeated seven times.

### Culture of two OES transformants and WT for methylomics, transcriptomics, and metabolomics.

A modified CMB (20 g corn, 10 g glucose, 0.4 g tryptone, 0.4 g vitamin B_1_, and 1L H_2_O) was used to culture the mycelia of transformants SA4 and SA6, and the WT of S. hirsutum, which were then grown at 28°C on a rotatory shaker (180 rpm) for 10 and 15 days. The mycelia were separated by filtering, removing the extra water by drying, and then frozen by liquid nitrogen and stored at −80°C. The mycelia samples were used for methylomics and transcriptomics. Another group samples were cultured in PDB (20 g potato, 20 g glucose, and 1L H_2_O) and used for transcriptomics. The sequences and data analysis of methylomics and transcriptomics were performed by Beijing Biomarker Technology Company (Beijing, China).

We determined the methylation status of each CpG site on each read using Oxford Nanopore Technologies with the hidden Markov model (54). Tomb was used to detect CHH (H = A/T/C), CHG, and 6mA sites (55). The current signal data generated from the nanopore read is called the waveform, and the re-squiggle algorithm refers to the distribution of the current signal waveform to the reference sequence. The Tomb software first re-squiggles the sequencing data. De novo, Alternative Model, and Sample Compare are available to select; two methods are used to detect non-classical bases. In the present study, a more accurate Alternative Model was used to detect CHH, CHG, and 6mA sites (55). The methylation level of the sites with high sequencing depth is more real, so C-sites above 10X depth and A-sites above 25X depth are reserved for subsequent analysis. In all cases, hyper- and hypomethylation were defined relative to the levels in the control sample.

To identify DEGs, each gene transcript was calculated with the TPM method. RNA-Seq by expectation maximization was used to quantify gene abundance. KEGG analyses were performed to enrich DEGs to the GO terms of function classes and metabolic pathways at the significance of Bonferroni-corrected *P < *0.05, in comparison to the whole-transcriptome background. We also classified all the DEGs, and selected secondary metabolism related genes, including cytochrome P450\PKS\terpene for the heatmap.

The mycelium of S. hirsutum (WT and 2 OES transformants) were cultured on PDA for 7 days, then inoculated in PDB (300 mL broth in 500-mL Erlenmeyer flask), and cultured in the dark at 28°C and 180 rpm. The cultures were collected from each experimental group at 14 and 21 days, respectively. The mycelium was removed by filtering through 2 layers of gauze (the hypha of each bottle was dried, and the dry weight was recorded), and the broth was extracted with an equal volume of ethyl acetate 5 times; then, the extract was concentrated, evaporated to dryness, and fully dissolved in 2 mL methanol, which was filtered to remove insoluble residue. Finally, the samples in methanol were diluted proportionally according to the mycelium dry weight (in the same culture period, the dilution ratio of the other samples is the dry weight of mycelium/dry weight of the least mycelium).

### Metabolomic data acquisition and statistical analysis.

Untargeted LC-MS metabolomics was performed on a Dionex UltiMate 3000 LC system coupled with a Q Exactive Orbitrap mass spectrometer (Thermo). All samples were separated on a Hypersil Gold column (100 mm × 2.1 mm, Thermo Fisher Scientific) with a particle size of 1.9 μm at an LC flow rate of 300 μL/min and a column temperature of 40°C. Mobile phase A was 0.1% formic acid in water, and mobile phase B was 0.1% formic acid in methanol. The 30 min gradient for positive ESI mode was set as follows: 0 to 2 min, 5% solvent B; 2 to 22 min, 5 to 95% solvent B; 22 to 25 min, 95% solvent B; and 25 to 30 min, 5% solvent B. The injection volume was 5 μL, and each sample was injected in triplicates. The injection order was randomized, and the group information was blinded for LC–MS analysis. The instrument settings were as follows: capillary temperature of 350°C, sheath gas flow rate of 35 (arbitrary units), auxiliary gas flow rate of 8 (arb), spray voltage of 3.5 kV, full MS resolution of 70,000, and MS/MS resolution of 17,500. Each sample was prepared in biological triplicate. The LC–MS instrument was controlled using Thermo Scientific Xcalibur 4.1 software.

The raw data file was Compound Discoverer (CD version 3.1, Thermo Fisher Scientific) software for metabolomics data analysis. A blank sample was used for background subtraction and noise removal during the pre-processing step. The data were analyzed in 3 groups (SA4, SA6, and WT). The QC samples were used to normalize each individual compound and compensate for instrumental drift. For analysis of the data on metabolite variation in the 3 groups, simple univariate statistical analyses were carried out on log_2_-transformed data using a paired *t* test. Volcano plots were created using these data, with a threshold of *P < *0.05 and absolute log_2_ fold change of >2 set for defining a notable change in compound abundance between WT and OES transformants. All components were searched against an accurate mass database consisting of known fungal metabolites using a mass tolerance of 10 ppm. The database was prepared using SciFinder, and additional fungal natural products were found in the literatures. Meanwhile, other potential compound identifications were obtained by comparing the MS/MS scan with the MZCloud, ChemSpider, and MZvault libraries. To confirm and evaluate intact mass-based identifications, manual analyses of fragmentation data were performed as described below. All compounds tentatively identified via accurate intact mass were confirmed using accurate mass, tandem MS (MS^2^) data. To ensure that low-quality spectra were not included, MS^2^ spectra containing fewer than 5 peaks at >1% relative abundance were excluded from analysis. Additionally, spectra containing more than 100 peaks at >1% abundance were included only if >20% of the peaks appeared in the higher *m/z* half of the spectrum. Both general fragmentation rules and fragmentation library modes were used. When published fragmentation was available, a comparison was also performed to further confirm identifications.

### Extraction, isolation, and spectroscopic data.

The mycelium of OES SA6 transformants were cultured on PDA for 7 days, then inoculated in PDB (20 L, 400 mL broth in 1 L Erlenmeyer flask), and cultured in the dark at 28°C and 180 rpm. The cultures were collected at 21 days. The broth was extracted by ethyl acetate five times to obtain the total crude extract (6.6 g). During the subsequent process of isolation and purification, the target compounds were monitored using LC–MS as a guide to indicate purity. The EtOAc extract (6.6 g) was purified on a silica gel column (200 to 300 mesh) using a petroleum ether–acetone (10:1 to 6:4) gradient first and then CHCl_3_–MeOH (10:1 to 0:100) to yield 10 fractions (Fr.1 to Fr.10). Fr.4 (0.931g) was chromatographed on a column of silica gel using gradient-elution petroleum ether/EtOAc (from 100:0 to 10:1), and then purified using Sephadex LH-20 (acetone) to obtain metabolite 1 (2 mg). Fr.6 (1.07 g) was separated on a column of silica gel (200 to 300 mesh) eluted with a CHCl_3_–MeOH gradient solvent system (100:2 to 6:4) and was then purified using Sephadex LH-20 (MeOH) to obtain compound 2 (2 mg).

Sterenin N (1): Colorless amorphism; [α]23 D + 7.78 (*c* 0.09, MeOH); UV (MeOH) *λ*_max_ (log ε) 197 (4.33), 220 (3.88), 284 (3.43) nm; ^1^H NMR (CDCl_3_, 600 MHz) and ^13^C NMR (CDCl_3_, 150 MHz) data (Table S11); positive ESI-MS *m/z* 359 [M+H]^+^, 381 [M + Na]^+^; HR-ESI-MS *m/z* 381.16700 [M + Na]^+^ (calcd for C_21_H_26_O_5_Na, 381.16697).

Sterenin O (2): Colorless amorphism; [α]23 D + 131.00 (*c* 0.10, MeOH); UV (MeOH) *λ*_max_ (log ε) 196 (4.80), 264 (4.28), 303 (3.84) nm; ^1^H NMR (CD_3_OD, 600 MHz) and ^13^C NMR (CD_3_OD, 150 MHz) data (Table S11); positive ESI-MS *m/z* 497 [M + Na]^+^; HR-ESI-MS *m/z* 497.17749 [M + Na]^+^ (calcd for C_25_H_30_O_9_, 497.17820).
