# Supplementary material for: Identification and Mechanism of Action of the Global Secondary Metabolism Regulator SaraC in Stereum hirsutum
Source: Microbiol Spectr. 2022 Nov 21;10(6):e02624-22. doi: 10.1128/spectrum.02624-22 (PMC9769804; doi:10.1128/spectrum.02624-22)
Supplement: Supplemental file 1 — Fig. S1 to S19 and Tables S1 to S4 and S6 to S11. Download spectrum.02624-22-s0001.pdf, PDF file, 1.3 MB [file spectrum.02624-22-s0001.pdf]

Supplementary materials for

Identification and mechanism of action of the global secondary metabolism regulator SaraC in *Stereum hirsutum*

Qian-Yi Hu[#], Xue-Juan Pu[#], Guo-Hong Li, Chun-Qiang Li, Hong-Mei Lei, Ke-Qin Zhang, Pei-Ji Zhao[*]

State key Laboratory for Conservation and Utilization of Bio-Resources in Yunnan, School of Life Sciences, Yunnan University, Kunming, Yunnan 650091, China.

[#]These authors contributed equally: Qian-Yi Hu, Xue-Juan Pu

*Correspondence and requests for materials should be addressed to Pei-Ji Zhao (email: pjzhao@ynu.edu.cn).

Content

1. **Figures S1-S19.**
2. **Tables S1-S4, S6 -S11.**

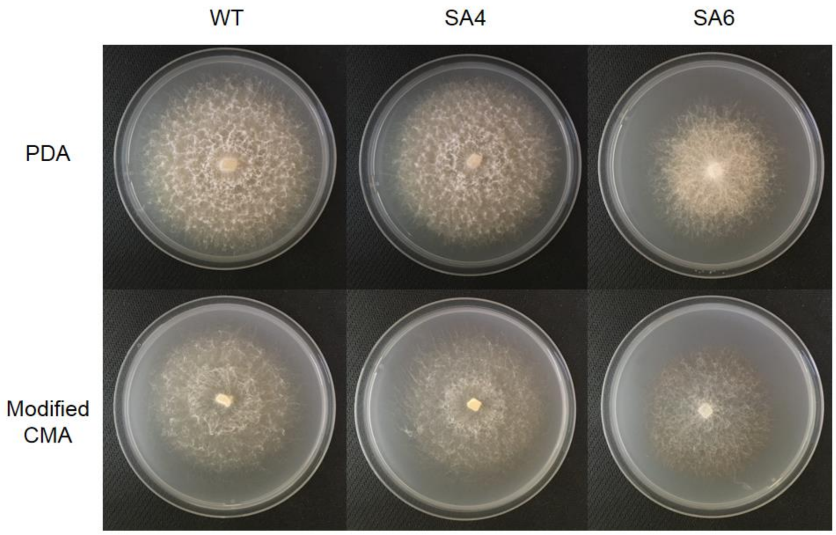

Figure S1 The colony of two transformants compared with the WT grown on two medium for 7d.

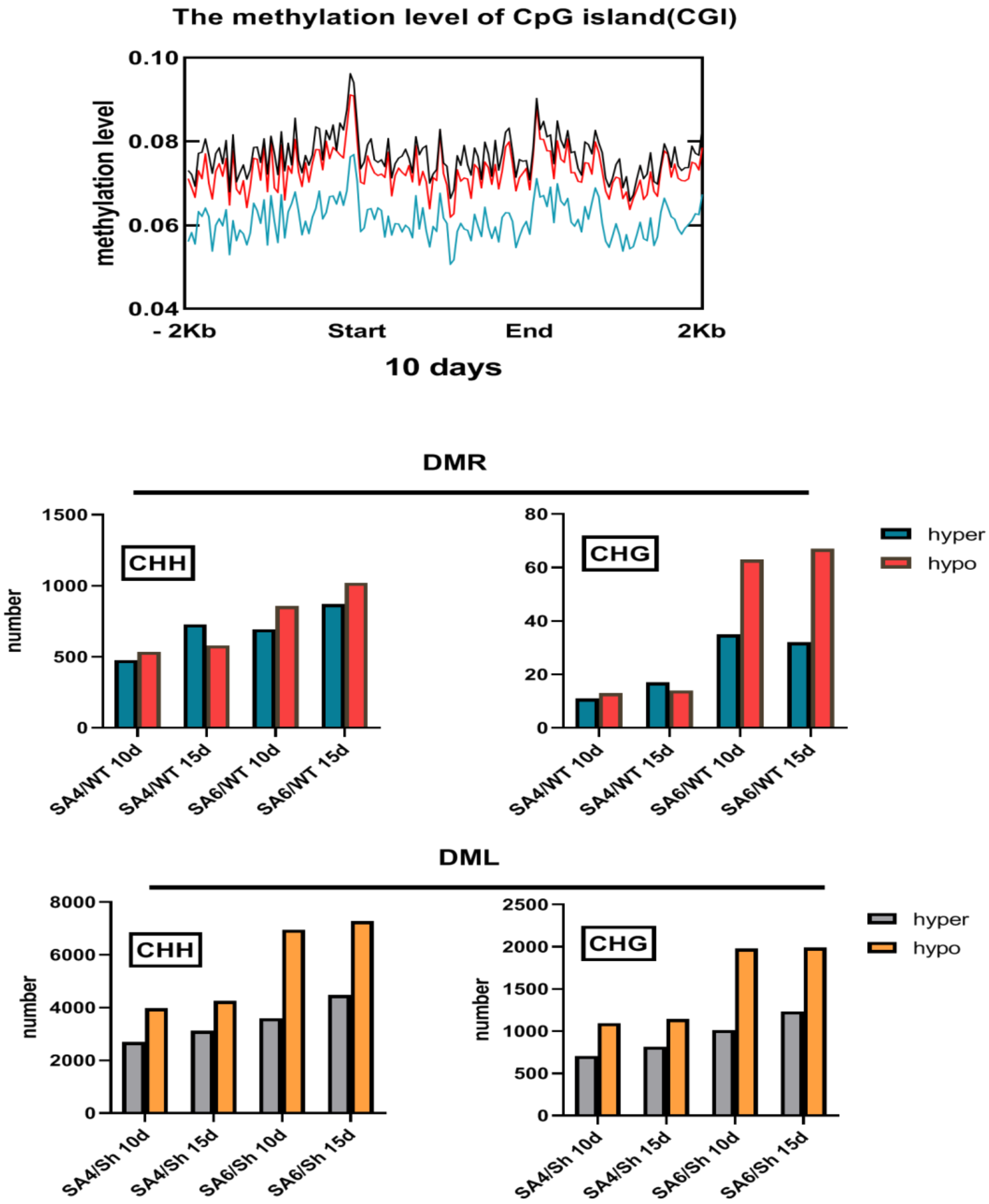

Figure S2 The methylation level of CpG island and The DMR/DML of CHH and CHG.

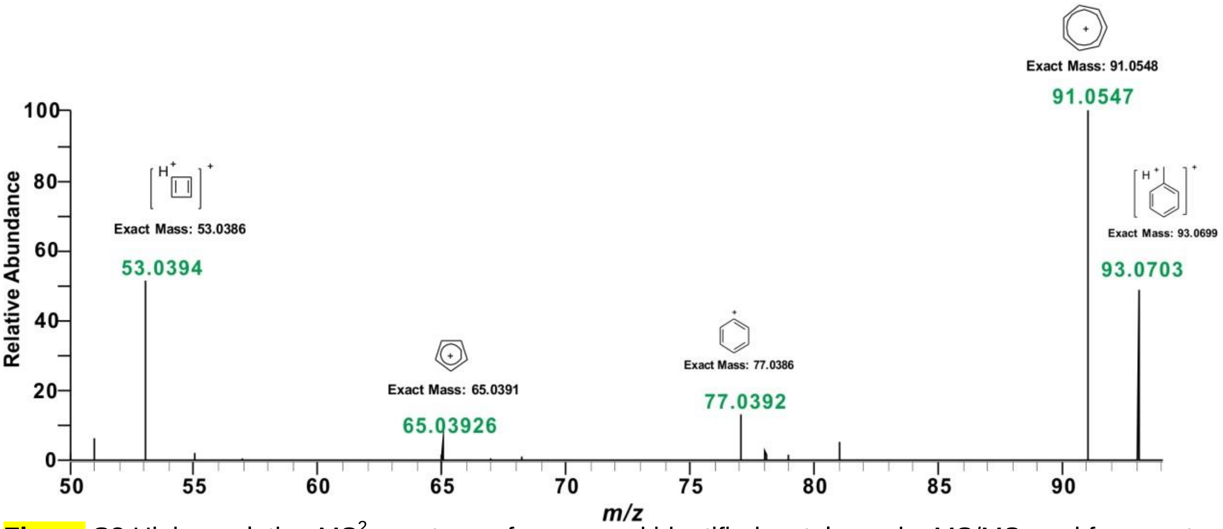

Figure S3 High resolution MS^2 spectrum of compound identified as toluene by MS/MS, and fragments explained the major fragment ions.

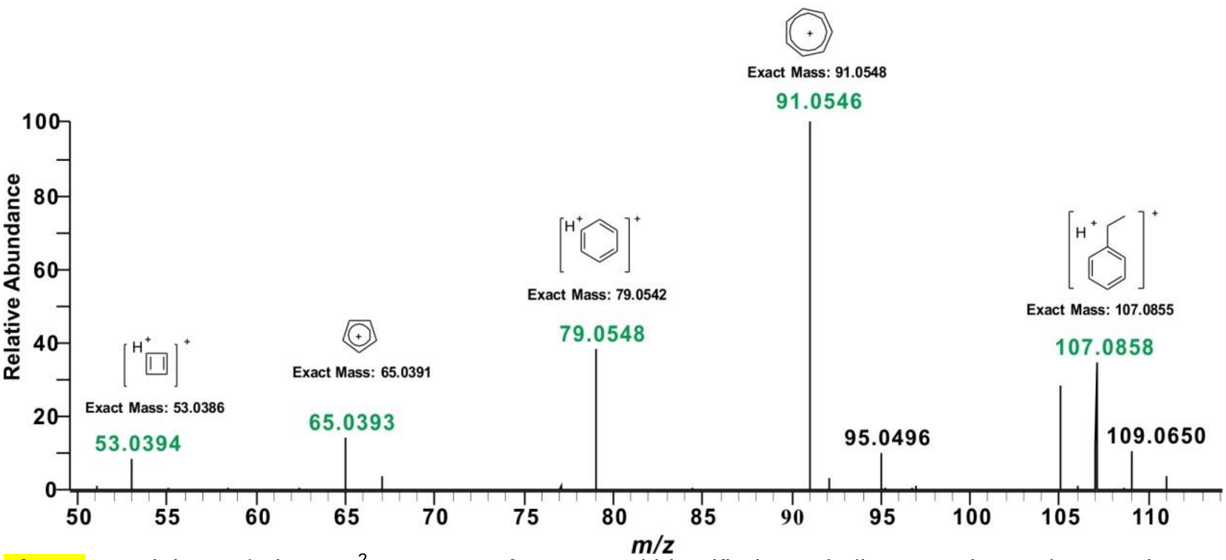

Figure S4 High resolution MS^2 spectrum of compound identified as ethylbenzene by MS/MS, and fragments explained the major fragment ions.

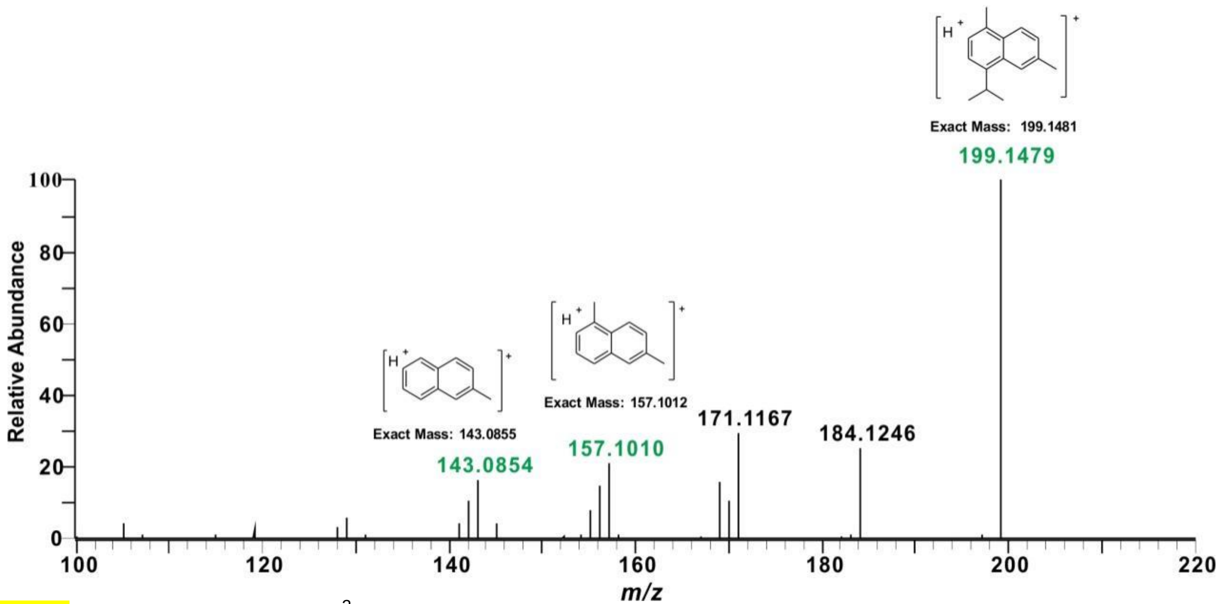

Figure S5 High resolution MS^2 spectrum of compound identified as cadalene by MS/MS, and fragments explained the major fragment ions.

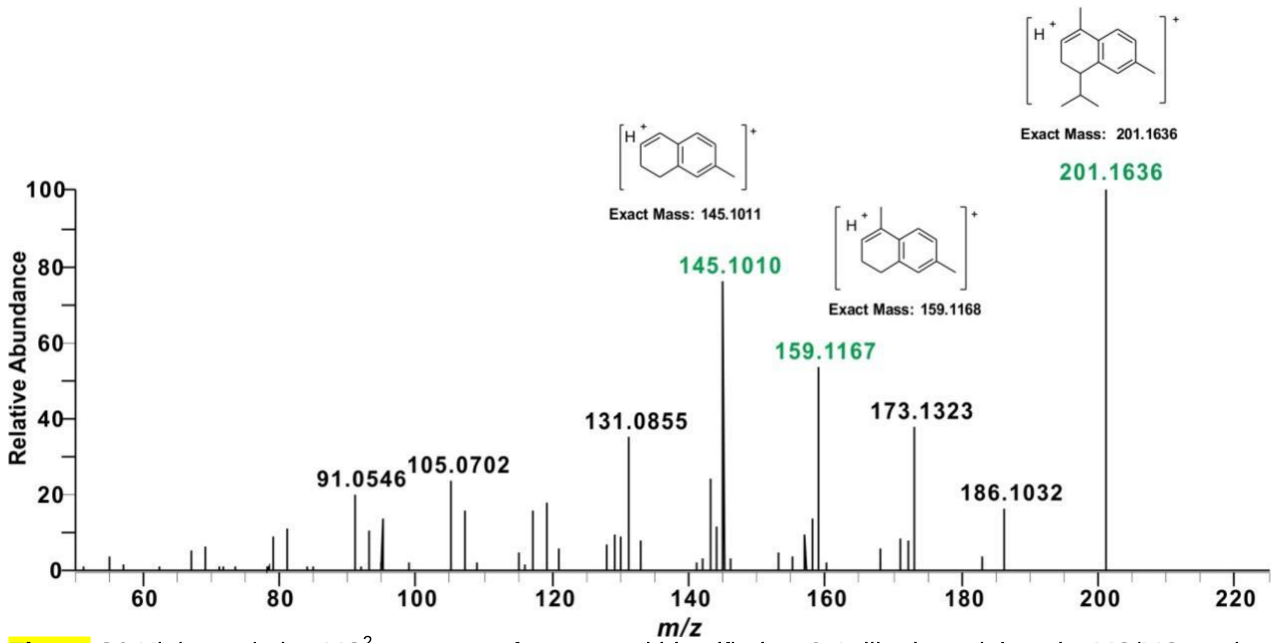

Figure S6 High resolution MS^2 spectrum of compound identified as 3,4-dihydrocadalene by MS/MS, and fragments explained the major fragment ions.

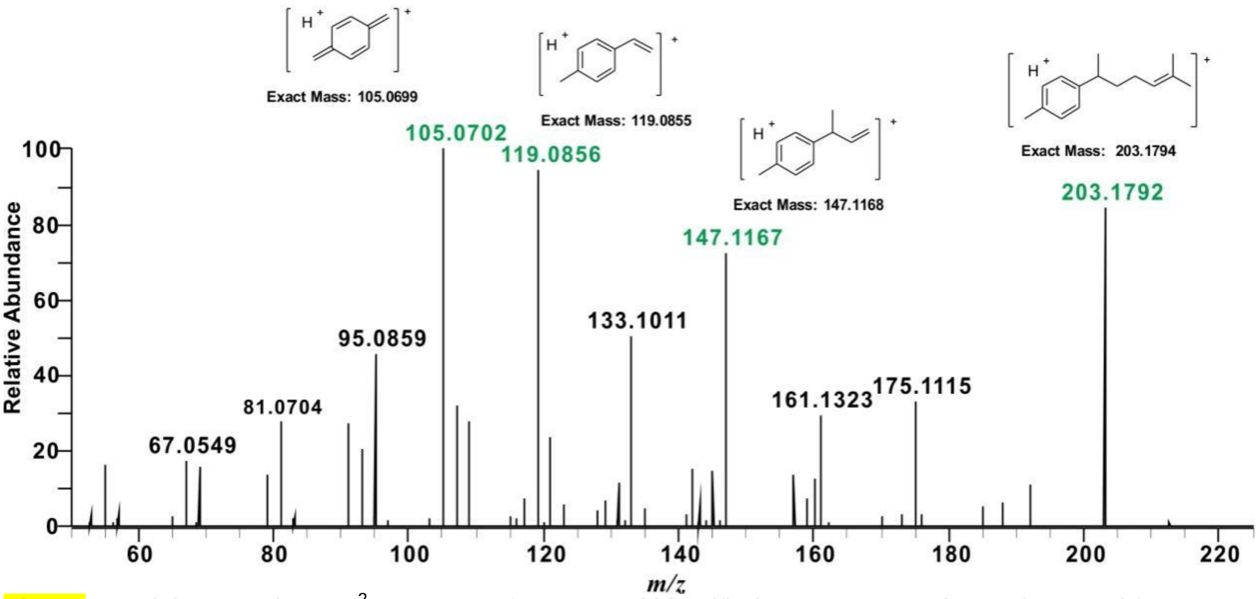

Figure S7 High resolution MS^2 spectrum of compound identified as curcumene by MS/MS, and fragments explained the major fragment ions.

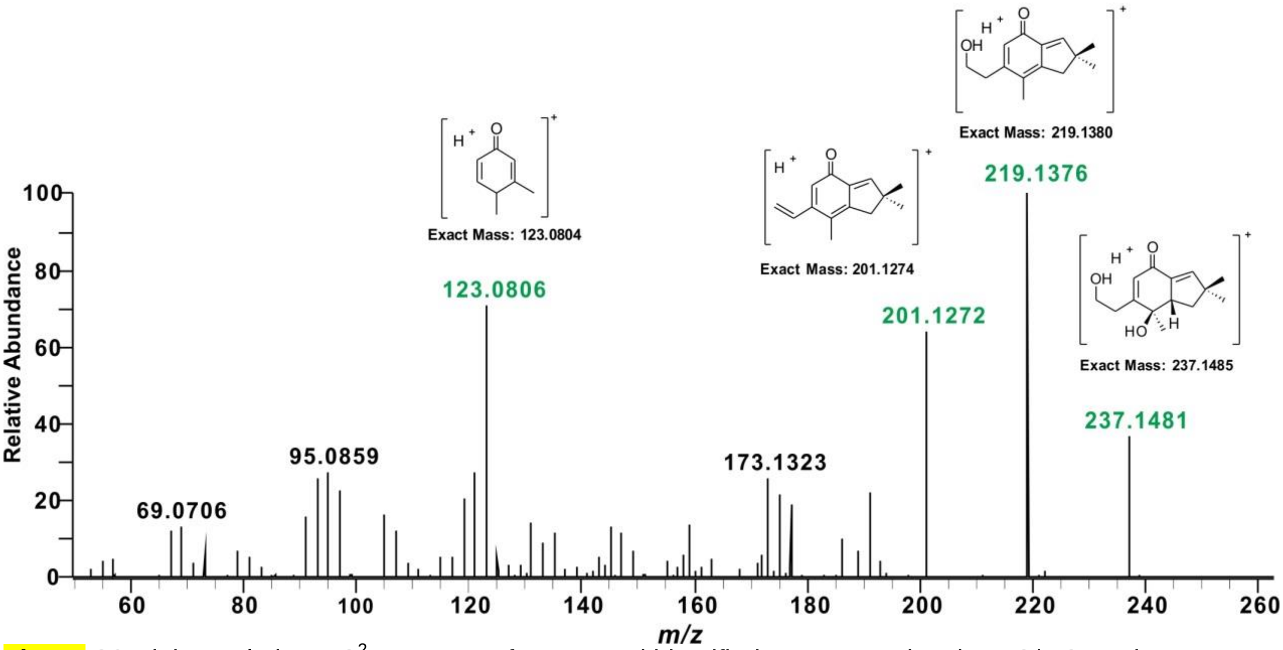

Figure S8 High resolution MS^2 spectrum of compound identified as sterostrein L by MS/MS, and fragments explained the major fragment ions.

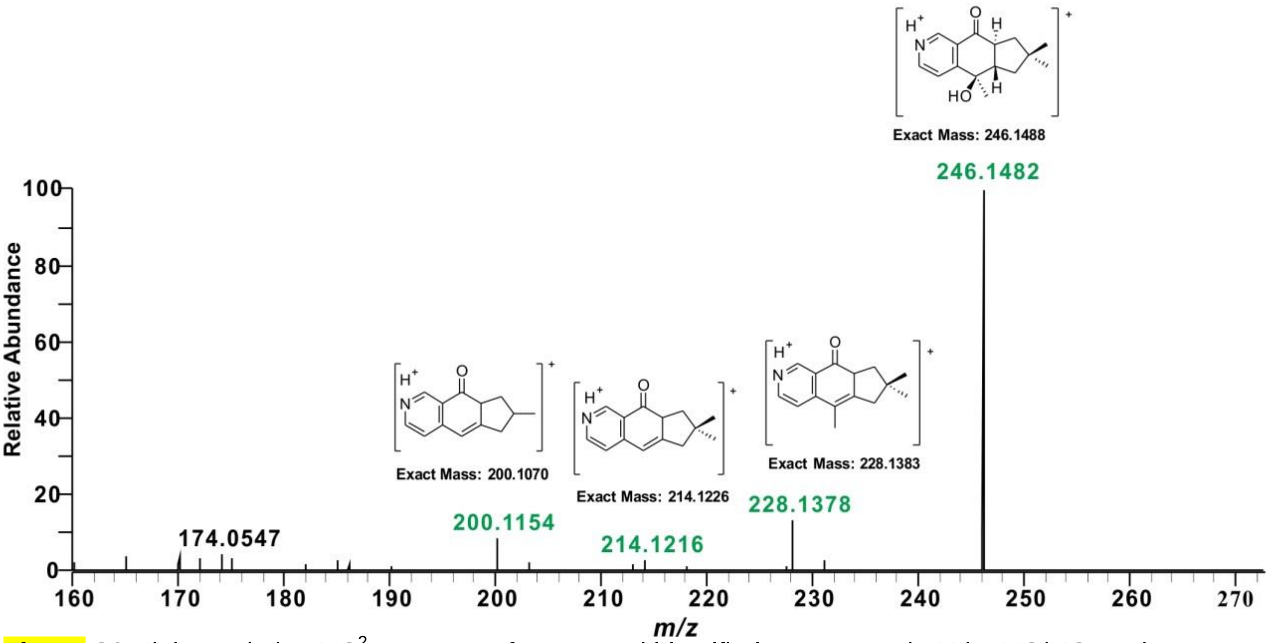

Figure S9 High resolution MS^2 spectrum of compound identified as sterostrein M by MS/MS, and fragments explained the major fragment ions.

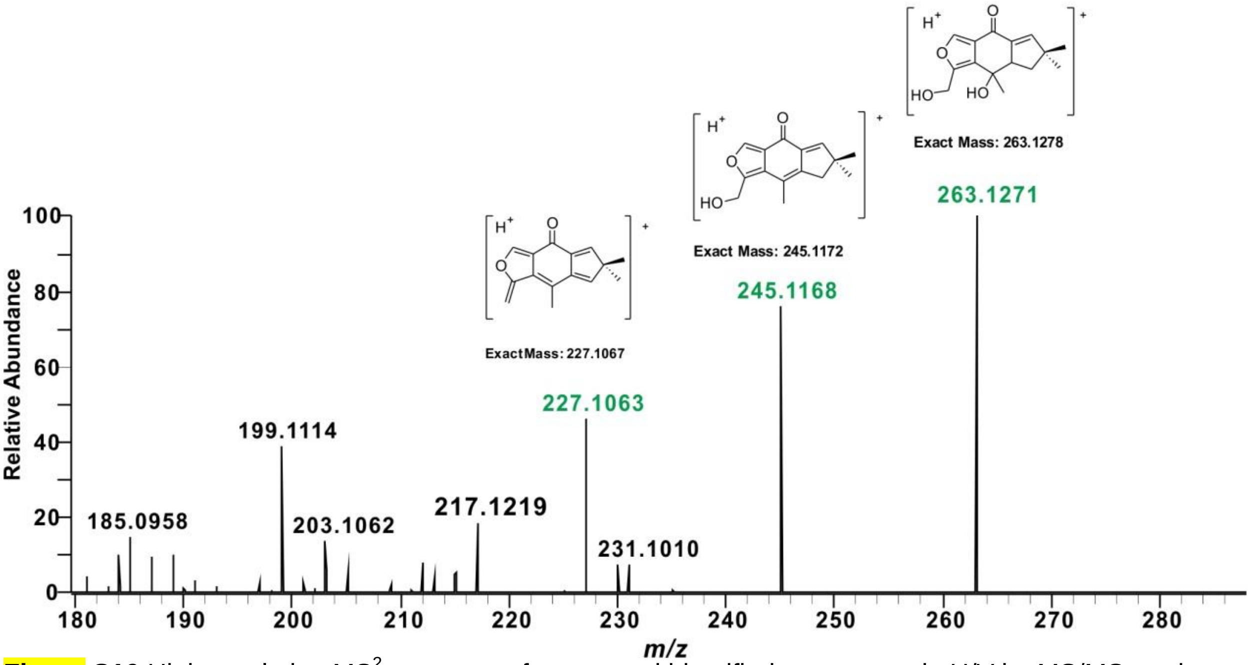

Figure S10 High resolution MS^2 spectrum of compound identified as sterostrein H/V by MS/MS, and fragments explained the major fragment ions.

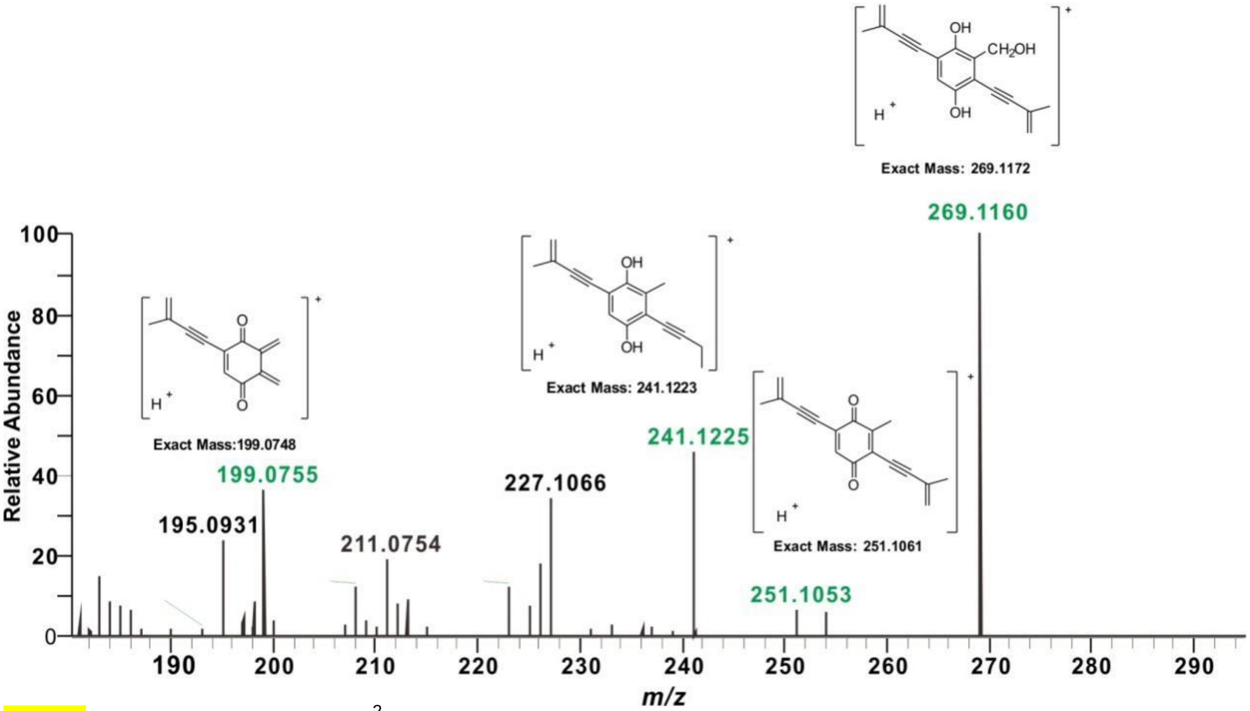

Figure S11 High resolution MS^2 spectrum of compound identified as 3-(hydroxymethyl)-2,5-bis(3-methylbut-3-en-1-ynyl)benzene-1,4-diol by MS/MS, and fragments explained the major fragment ions.

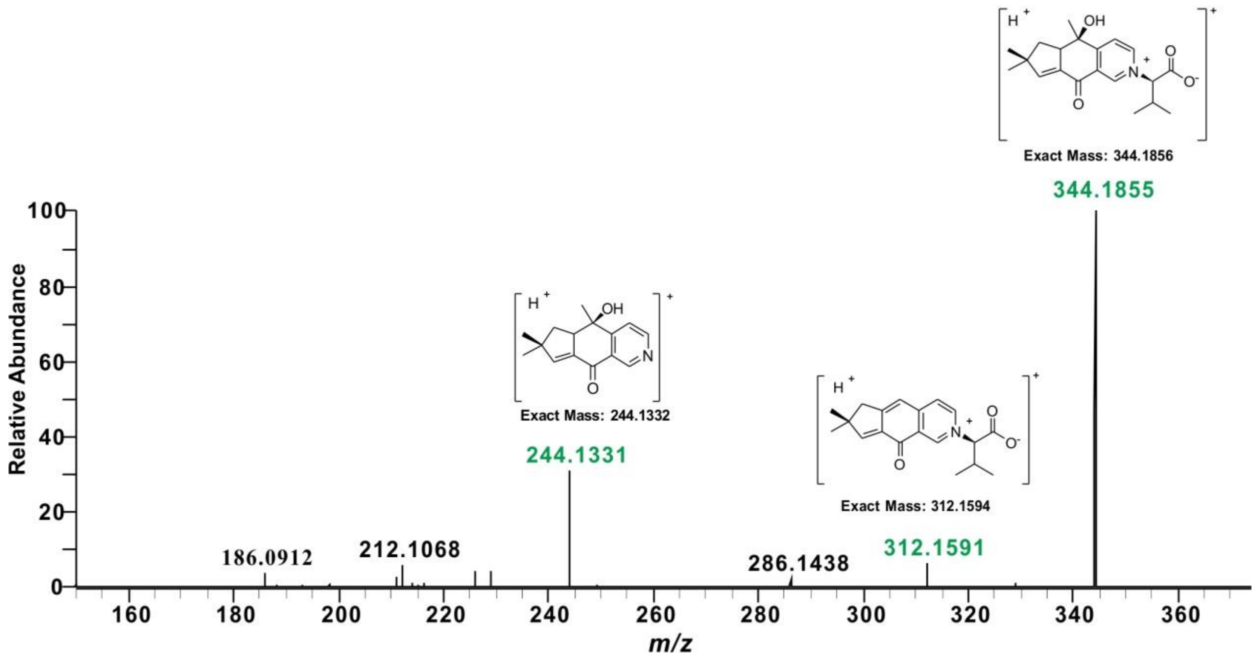

Figure S12 High resolution MS^2 spectrum of compound identified as stereumamide C by MS/MS, and fragments explained the major fragment ions.

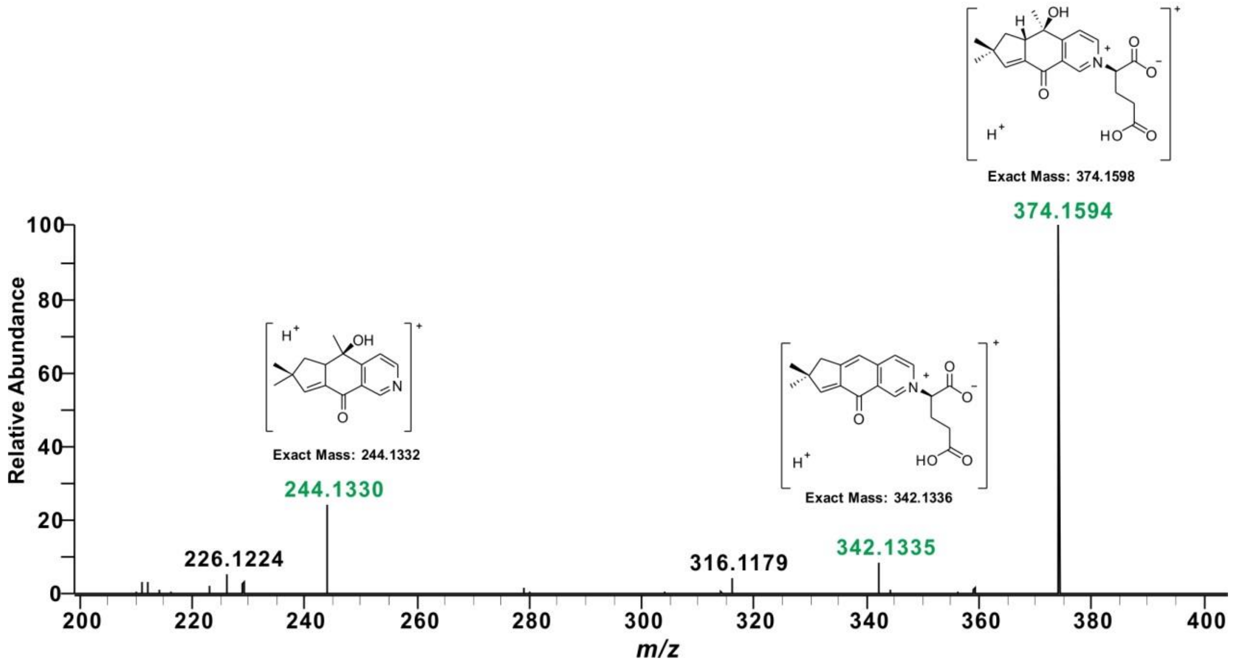

Figure S13 High resolution MS^2 spectrum of compound identified as stereumamide I by MS/MS, and fragments explained the major fragment ions.

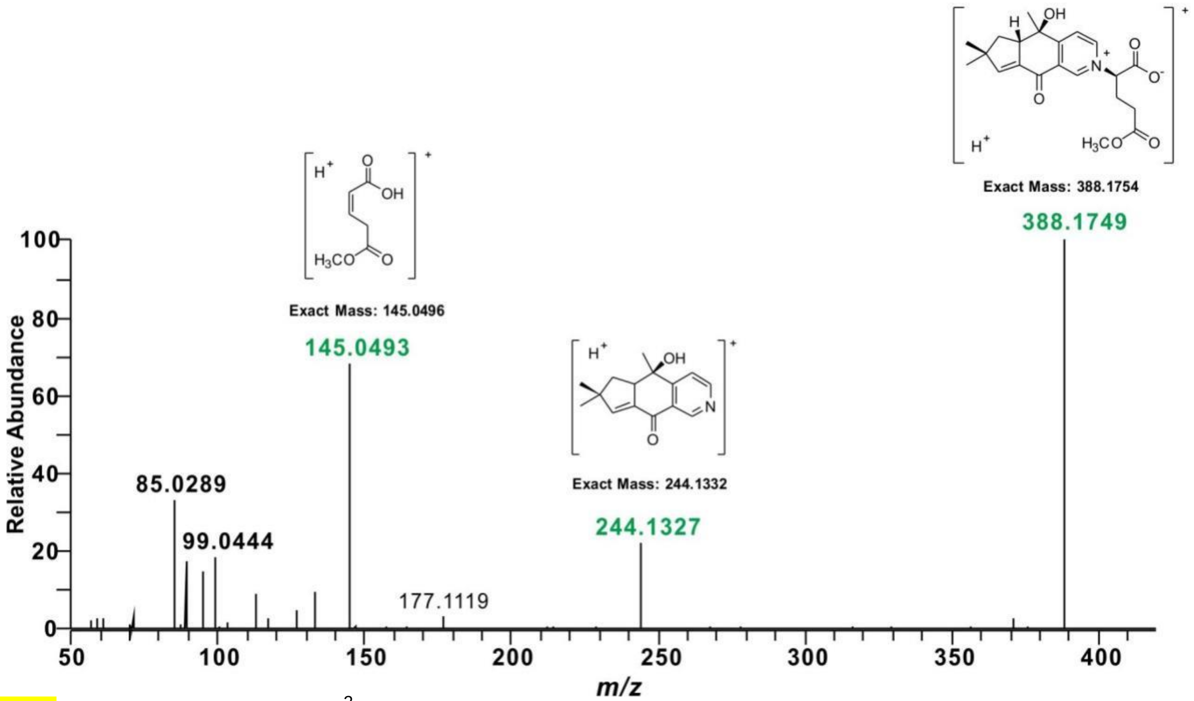

Figure S14 High resolution MS^2 spectrum of compound identified as stereumamide F by MS/MS, and fragments explained the major fragment ions.

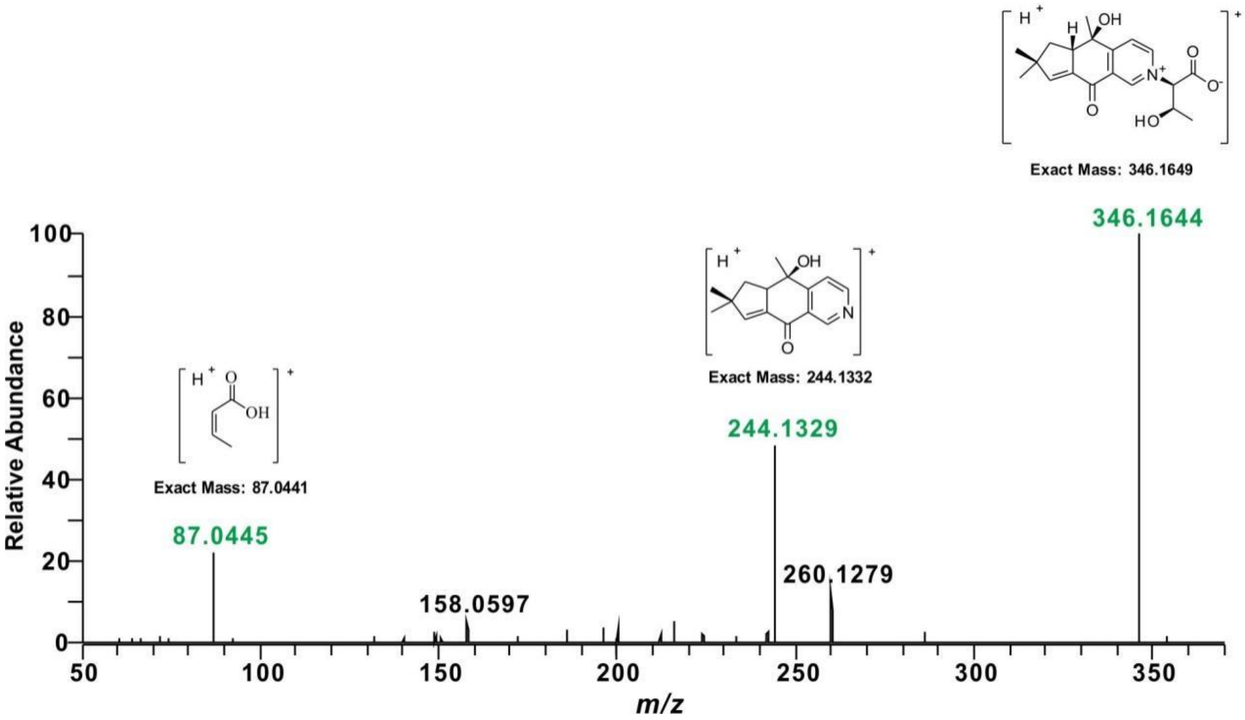

Figure S15 High resolution MS^2 spectrum of compound identified as stereumamide A by MS/MS, and fragments explained the major fragment ions.

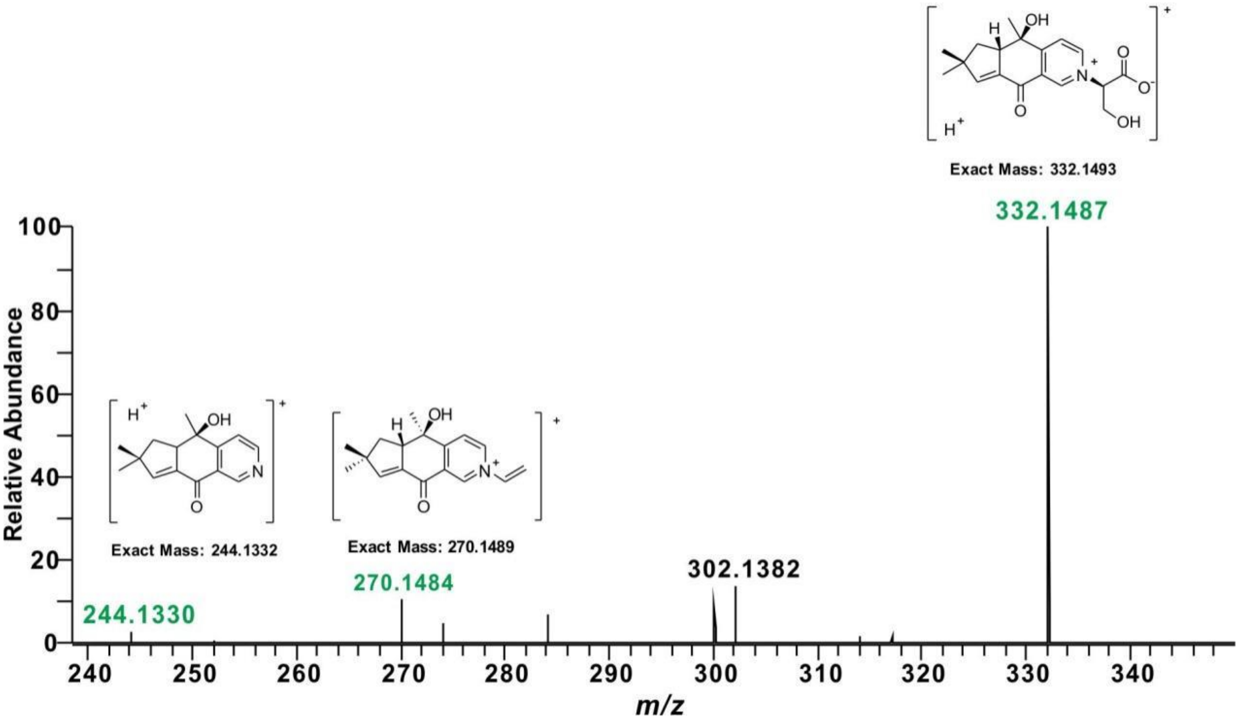

Figure S16 High resolution MS^2 spectrum of compound identified as stereumamide G by MS/MS, and fragments explained the major fragment ions.

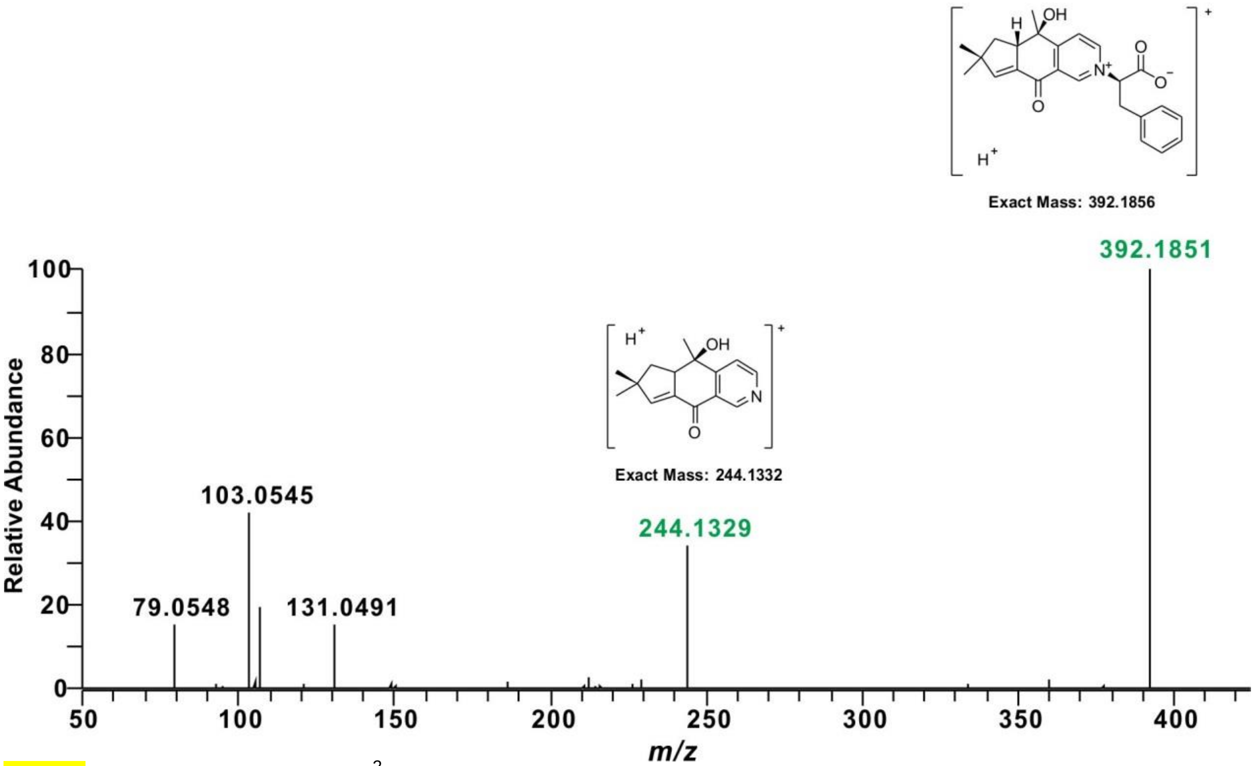

Figure S17 High resolution MS^2 spectrum of compound identified as stereumamide K by MS/MS, and fragments explained the major fragment ions.

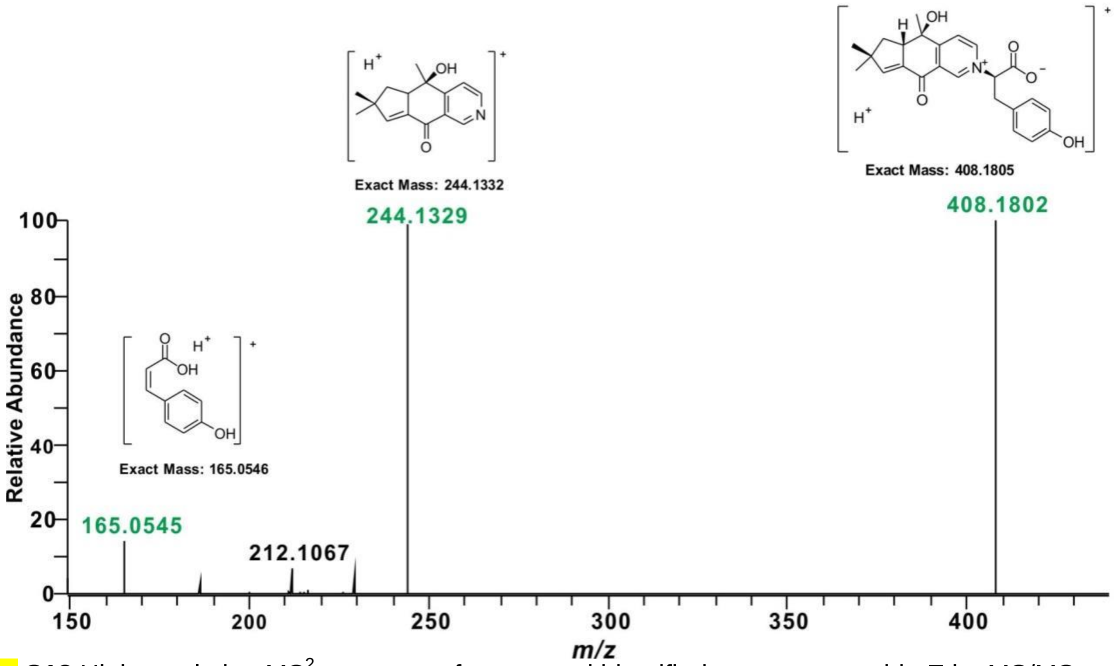

Figure S18 High resolution MS^2 spectrum of compound identified as stereumamide E by MS/MS, and fragments explained the major fragment ions.

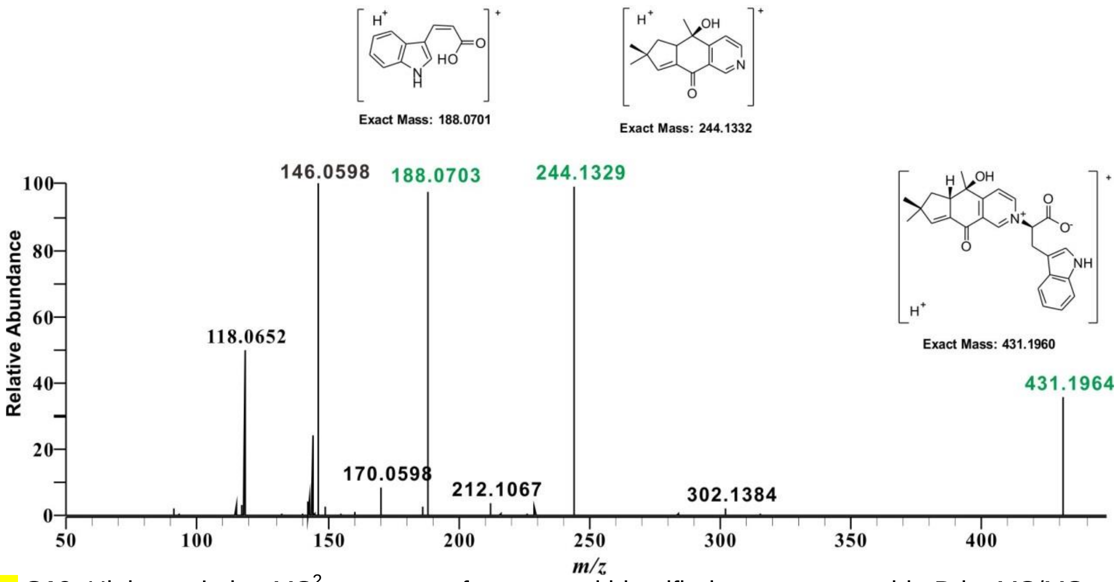

Figure S19. High resolution MS^2 spectrum of compound identified as stereumamide D by MS/MS, and fragments explained the major fragment ions.

Table S1 The KD values of the interactions of oligonucleotide with SaraC protein

Name	Sequence	Molecular weight (Da)	ka (1/Ms)	kd (1/s)	KD (M)
Oligo1309	ATCCAG^{m}**A**GCG^{m}**A** GCCTATTACG	6443	2.093×10^{4}	0.03500	1.672×10^{-6}
Oligo5564	CGATCG^{m}**G**ATTT^{m}**G** A**C**GGTT	5573	2.756×10^{4}	0.02262	8.208×10^{-7}
Oligo7291	GATT^{m}**C**CTTC	2679	2.821×10^{5}	0.1869	6.624×10^{-7}

Table S2 Clean reads data tables of methylome

Sample	SeqNum	SumBase	N50	N90	MeanLen	MaxLen	MeanQual
SH-10	1,389,650	11,092,078,397	9,399	5,333	7,981	129,358	9.73
SA4-10	1,386,731	10,805,495,005	9,153	5,171	7,792	93,238	9.72
SA6-10	1,325,657	10,375,284,836	9,035	5,204	7,826	88,610	9.70
SH-15	1,225,933	9,494,302,296	9,138	5,211	7,744	85,414	9.63
SA4-15	1,080,082	9,298,981,563	10,098	5,759	8,609	96,005	9.56
SA6-15	1,285,325	10,175,871,225	9,649	5,216	7,916	106,535	9.66

Note: SeqNum: Sequence number; **SumBase:** Total base number; **N50：** Data N50 length; **N90:** Data N90 length; **MeanLen:** Average length of reads; **MaxLen:** The Max Reads length; **MeanQual:** Average Reads quality value.

Table S3 Average methylation level of four methylation sites in the whole genome at 10 and 15 days

	10 d (%)				15 d (%)			
	CpG	CHG	CHH	6mA	CpG	CHG	CHH	6mA
WT	15.23	20.25	23.80	13.74	15.15	20.31	23.85	13.62
SA4	14.42	20.70	24.39	16.34	14.29	20.88	24.78	15.69
SA6	12.00	20.52	24.28	15.87	11.94	20.05	23.92	13.57

Table S4 Statistics of different types of methylation sites that above sequencing depth

Sample	6mA	CHG	CHH	CpG
WT-10	10,719,698	3,667,034	11,595,341	5,824,354
SA4-10	13,808,052	3,788,894	11,998,393	5,821,265
SA6-10	13,187,325	3,767,729	11,928,247	5,803,513
WT-15	10,294,790	3,659,798	11,571,213	5,807,281
SA4-15	12,568,168	3,727,654	11,795,493	5,800,016
SA6-15	10,289,390	3,643,682	11,518,653	5,804,639

Table S6. Methylation and transcription of genes in Cluster 2.4

Gene	Methylation	Transcription	Annotion
STEHIDRAFT_48573	N	-0.373	Glycosyl hydrolase family 79
STEHIDRAFT_49054	N	0.180	PLP-dependent transferase
STEHIDRAFT_50247	CpG	0.189	Gamma-glutamyl cyclotransferase
STEHIDRAFT_165595	N	0.260	None
STEHIDRAFT_73029	N	2.049	terpenoid synthase
STEHIDRAFT_49597	N	0.060	Aldo/keto reductase
STEHIDRAFT_118344	N	3.236	alcohol oxidase
STEHIDRAFT_91266	N	1.677	MFS general substrate transporter
STEHIDRAFT_50014	6mA	3.672	cytochrome P450
STEHIDRAFT_37206	N	--	None
STEHIDRAFT_118347	6mA	2.441	NAD-P-binding protein
STEHIDRAFT_91274	6mA	2.404	cytochrome P450
STEHIDRAFT_107839	6mA	1.474	aryl-alcohol-oxidase from pleurotus Eryingii
STEHIDRAFT_137017	N	2.885	FAD/NAD-P-binding domain-containing protein
STEHIDRAFT_118352	N	1.180	FAD-binding domain-containing protein
STEHIDRAFT_128191	N	2.035	Aldo/keto reductase
STEHIDRAFT_118353	N	2.342	cytochrome P450

Table S7 Statistics of differentially transcription genes in PDB

DEG Set	DEG Number	up-regulated (annotation)	down-regulated (annotation)
SA4-10	718	399 (163)	319 (143)
SA6-10	2,953	1,462 (511)	1,491 (601)
SA4-15	1,434	613 (187)	821 (444)
SA6-15	1,411	775 (207)	636 (345)

Note: The annotation is analyzed by GO/COG/KOG

Table S8 Forty-one known compounds confidently identified in OES transformants vs WT experiments

Compound names	Ion mode	Test mass(m/z)	Molecular formula	Theoretical mass (m/z)
Toluene	$[M + H]^+$	93.0703	C_7H_8	93.0699
Ethylbenzene	$[M + H^+$	107.0858	C_8H_{10}	107.0855
Cadalene	$[M + H]^+$	199.1479	$C_{15}H_{18}$	199.1481
3,4-dihydrocadalene	$[M + H]^+$	201.1636	$C_{15}H_{20}$	201.1638
Curcumene	$[M + H]^+$	203.1792	$C_{15}H_{22}$	203.1794
4'-hydroxy-3'-prenylaceto-phenone (HPP)	$[M + H]^+$	205.1222	$C_{13}H_{16}O_2$	205.1223
3-Hydroxy-6-methoxy-2-(3-methyl-3-buten-1-yn-1-yl)- benzene methanol (HMPBM)	$[M + H]^+$	219.1012	$C_{13}H_{14}O_3$	219.1016
2-(3-methyl-2-buten-1-yl)-4-methoxyethyl-phenol (PMP)	$[M + H]^+$	221.1533	$C_{14}H_{20}O_2$	221.1536
Stereumene B	$[M + H]^+$	231.1381	$C_{15}H_{18}O_2$	231.138
Vibradiol	$[M + H]^+$	235.1325	$C_{14}H_{18}O_3$	235.1329
Sterostrein L	$[M + H]^+$	237.1481	$C_{14}H_{20}O_3$	237.1485
sterostrein M	$[M + H]^+$	246.1487	$C_{15}H_{19}O_2N$	246.1489
Hirsutenol A	$[M + H]^+$	249.1480	$C_{15}H_{20}O_3$	249.1485
Hirsutenol B	$[M + H]^+$	251.1637	$C_{15}H_{22}O_3$	251.1642
Sterostrein I	$[M + H]^+$	253.1795	$C_{15}H_{24}O_3$	253.1798
Drimene-2,11-diol	$[M + Na]^+$	261.1822	$C_{15}H_{26}O_2$	261.1825
Sterostrein P	$[M + H]^+$	261.1119	$C_{15}H_{16}O_4$	261.1121
Sterostrein N	$[M + H]^+$	262.1439	$C_{15}H_{19}O_3N$	262.1438
Sterostrein H/V	$[M + H]^+$	263.1273	$C_{15}H_{18}O_4$	263.1278
Sterostrein D/E	$[M + H]^+$	265.1431	$C_{15}H_{20}O_4$	265.1434
6-acetoxymethyl-2,2-dimethyl-3,4-dihydro-2H-chromene-3,4-diol (ADMC)	$[M + H]^+$	267.1222	$C_{14}H_{18}O_5$	267.1227
Sydonic acid	$[M + H]^+$	267.1567	$C_{15}H_{22}O_4$	267.1591
3-(hydroxymethyl)-2,5-bis(3-methylbut-3-en-1-ynyl)benzene-1,4-diol (HBPB)	$[M + H]^+$	269.1168	$C_{17}H_{16}O_3$	269.1172
2,5-dihydroxy-3-isoprenyl-6-(3-methylbut-3-en-1-ynyl) benzaldehyde (DHBPBA)	$[M + H]^+$	271.1323	$C_{17}H_{18}O_3$	271.1329
Cheodontoin K	$[M + Na]^+$	275.1620	$C_{15}H_{24}O_3$	275.1618
Sterostrein F/G	$[M + H]^+$	281.1380	$C_{15}H_{20}O_5$	281.1384
Sterostrein S	$[M + H]^+$	295.1511	$C_{16}H_{22}O_5$	295.154
Sterostrein W	$[M + H]^+$	303.1955	$C_{19}H_{26}O_3$	303.1955

4'-hydroxy-6'-(3''-methyl-2''-butenyl)-phenyl-2,4-dihydroxy-6-methyl-benzoate (HPPDHMB)	$[M + H]^+$	329.1373	$C_{19}H_{20}O_5$	329.1384
Stereumamide C	$[M + H]^+$	344.1857	$C_{20}H_{25}O_4N$	344.1856
Stereumamide B/H	$[M + H]^+$	358.2007	$C_{21}H_{27}O_4N$	358.2013
Stereumamide I	$[M + H]^+$	374.1592	$C_{20}H_{23}O_6N$	374.1598
Stereumamide F	$[M + H]^+$	388.1750	$C_{21}H_{25}O_6N$	388.1755
MS-3	$[M+Na]^+$	411.1415	$C_{21}H_{24}O_7$	411.1414
Sterenin H	$[M + Na]^+$	425.1567	$C_{22} H_{26} O_7$	425.1571
stereumamide J	$[M + H]^+$	430.2218	$C_{24}H_{31}O_6N$	430.2224
Stereumamide A	$[M + H]^+$	346.1644	$C_{19}H_{23}O_5N$	346.1649
Stereumamide G	$[M + H]^+$	332.1488	$C_{18}H_{21}O_5N$	332.1493
Stereumamide K	$[M + H]^+$	392.1850	$C_{24}H_{25}O_4N$	392.1856
Stereumamide E	$[M + H]^+$	408.1800	$C_{24}H_{25}O_5N$	408.1806
Stereumamide D	$[M + H]^+$	431.1961	$C_{26}H_{26}O_4N_2$	431.1965

Table S9. Relative content of identified compounds in OES transformants vs WT experiments

Compound name	Ratio SA-6/WT	Ratio SA-4/WT	Compound name	Ratio SA-6/WT	Ratio SA-4/WT
Toluene*	10.57	0.8575	Sydonic acid	14.01	0.72
Ethylbenzene*	11.25	1.50	HBPB[a]*	14.01	25.34
Cadalene*	29.08	7.53	DHBPBA[a]	8.31	0.51
3,4-dihydrocadalene*	10.42	6.00	Cheodontoin K	68.29	6.28
Curcumene*	11.20	4.40	Sterostrein F/G	91.40	3.60
HPP[a]	35.95	17.24	Sterostrein S	264.06	7.70
HMPBM[a]	14.30	1.27	Sterostrein W	366.11	50.15
PMP[a]	15.95	3.71	HPPDHMB[a]	29.55	1.92
Stereumene B	11.76	9.82	Stereumamide C*	47.48	11.24
Vibradiol	76.40	7.43	Stereumamide B/H	40.56	7.59
Sterostrein L*	62.185	17.51	Stereumamide I*	758.28	140.78
sterostrein M*	10.57	3.72	Stereumamide F*	124.44	78.04
Hirsutenol A	21.37	3.17	MS-3	51.79	3.39
Hirsutenol B	25.24	22.92	Sterenin H	60.61	5.90
Sterostrein I	32.61	34.00	Stereumamide J*	75.17	64.90
Drimene-2,11-diol	8.26	3.11	Stereumamide A*	189.58	480.34
Sterostrein P	35.73	0.979	Stereumamide G*	59.94	440.50
Sterostrein N	155.16	88.37	Stereumamide K*	20.90	105.92
Sterostrein H/V*	299.29	43.56	Stereumamide E*	154.86	103.68
Sterostrein D/E	19.06	11.08	Stereumamide D*	47.52	54.66
ADMC[a]	19.06	0.40			

[a] The full compound names showed in the Supporting information Table S8
[*] Compounds marked with an asterisk (*) have associated MS/MS verification shown in the Supporting information.

Table S10. Relative content of unknown peaks in OES transformants vs WT experiments

m/z	Predict molecular formula	Theoretical molecular formula value	Ratio SA-4/WT	Ratio SA-6/WT	Retention time (min)
339.1585	$C_{21}H_{22}O_4$	339.1591	1.546	14.556	17.442
343.1510	$C_{20}H_{22}O_5$	343.1516	8.852	33.655	17.075
345.2032	$C_{21}H_{28}O_4$	345.2036	10.198	19.903	20.203
345.2032	$C_{21}H_{28}O_4$	345.2036	32.264	54.3057	19.164
361.1276	$C_{19}H_{20}O_7$	361.1282	2.772	126.071	13.437
375.1428	$C_{20}H_{22}O_7$	375.1438	2.536	54.546	15.365
375.1428	$C_{20}H_{22}O_7$	375.1438	9.836	29.924	16.995
379.1385	$C_{19}H_{22}O_8$	379.1387	1.112	24.628	8.739
381.1687	$C_{21}H_{26}O_5Na$	381.1672	1.520	37.672	16.165
385.1617	$C_{20}H_{26}O_6$	385.1622	2.087	25.365	19.14
401.1928	$C_{20}H_{26}O_7$	401.1935	1.3185	59.575	15.384
403.2108	$C_{23}H_{30}O_6$<br>$C_{21}H_{32}O_6Na$	403.2115<br>403.2091	1.755	62.280	20.56
439.1633	$C_{18}H_{31}O_{10}S$	439.1632	10.144	70.618	7.945
457.1824	$C_{23}H_{30}O_8Na$	457.1833	6.861	56.906	17.97
465.1907	$C_{25}H_{30}O_7Na$<br>$C_{27}H_{28}O_7$	465.1908<br>465.1884	5.150	23.539	17.881
467.2043	$C_{27}H_{30}O_7$	467.2040	5.919	32.211	21.524
497.1773	$C_{25}H_{30}O_9Na$	497.1782	0.089	10.208	16.296
543.1834	$C_{28}H_{30}O_{11}$	543.1837	1.143	21.688	14.67

Table S11. ^{1}H- (600 MHz) and ^{13}C-NMR (150 MHz) data for compounds **1** and **2**

No.	**1** (in $CDCl_3$)		No.	**2** (in CD_3OD)	
	δ_H (mult, J in Hz)	δ_C		δ_H (mult, J in Hz)	δ_C
1		125.9, s	1	-	141.1, s
2	6.63 (1H, s)	130.9, d	2	7.30 (1H, brs, 2.2)	130.0, d
3	-	126.4, s	3	-	134.9, s
4	-	153.2, s	4	-	149.1, s
5	6.58 (1H, d, 8.7)	115.3, d	5	7.09 (1H, d, 8.2)	123.5, d
6	6.64 (1H, d, 8.7)	128.3, d	6	7.28 (1H, dd, 8.2, 2.2)	126.9, d
7	3.22 (2H, s)	42.1, t	7	4.61 (2H, m)	64.7, t
8	3.24 (2H, m)	29.2, t	8	3.29 (2H, brs)	30.6, t
9	5.24 (1H, t, 7.3)	121.6, d	9	5.17 (1H, m)	123.0, d
10	-	134.6, s	10	-	133.9, s
11	1.73 (3H, s)	17.8, q	11	1.53 (3H, s)	17.7, q
12	1.77 (3H, s)	25.8, q	12	1.62 (3H, s)	25.8, q
13	-	80.4, s	1′	-	108.0, s
14		168.4, s	2′	-	163.4, s
15	5.43 (1H, s)	104.9, d	3′	6.60 (1H, m)	103.1, d
16	-	186.9, s	4′		165.8, s
17	5.43 (1H, s)	104.9, d	5′	6.63 (1H, m)	113.6, d
18	-	168.4, s	6′	-	144.2, s
13-OCH_3	3.15 (3H, s)	52.7, q	7′	-	171.4, s
14-OCH_3	3.80 (3H, s)	55.9, q	9′	2.62 (3H, s)	24.3, q
18-OCH_3	3.80 (3H, s)	55.9, q	1″	5.71 (1H, brd, 4.4)	101.6, d
5-OH	5.01 (1H, s)	-	2″	4.23 (1H, m)	73.5, d
			3″	4.10 (1H, m)	71.1, d
			4″	4.13 (1H, m)	88.0, d
			5″	3.66 (1H, m)	63.1, t
				2.73 1H, m)	
